# Strengthening Intraframework Interaction within Flexible MOFs Demonstrates Simultaneous Sieving Acetylene from Ethylene and Carbon Dioxide

**DOI:** 10.1002/advs.202207127

**Published:** 2023-01-26

**Authors:** Fang Zheng, Rundao Chen, Ying Liu, Qiwei Yang, Zhiguo Zhang, Yiwen Yang, Qilong Ren, Zongbi Bao

**Affiliations:** ^1^ Key Laboratory of Biomass Chemical Engineering of the Ministry of Education College of Chemical and Biological Engineering Zhejiang University 38 Zheda Road Hangzhou 310027 P. R. China; ^2^ Institute of Zhejiang University‐Quzhou 99 Zheda Road Quzhou Zhejiang Province 324000 China

**Keywords:** flexibility, gate‐opening, intraframework interaction, molecular sieve

## Abstract

Efficient separation of acetylene (C_2_H_2_)/ethylene (C_2_H_4_) and acetylene/carbon dioxide (CO_2_) by adsorption is an industrially promising process, but adsorbents capable of simultaneously capturing trace acetylene from ethylene and carbon dioxide are scarce. Herein, a gate‐opening effect on three isomorphous flexible metal–organic frameworks (MOFs) named Co(4‐DPDS)_2_MO_4_ (M = Cr, Mo, W; 4‐DPDS = 4,4‐dipyridyldisulfide) is modulated by anion pillars substitution. The shortest CrO_4_^2−^ strengthens intraframework hydrogen bonding and thus blocks structural transformation after activation, striking a good balance among working capacity, separation selectivity, and trace impurity removal of flexible MOFs out of nearly C_2_H_2_/C_2_H_4_ and C_2_H_2_/CO_2_ molecular sieving. The exceptional separation performance of Co(4‐DPDS)_2_CrO_4_ is confirmed by dynamic breakthrough experiments. It reveals the specific threshold pressures control in anion‐pillared flexible materials enabled elimination of the impurity leakage to realize high purity products through precise control of the intraframework interaction. The adsorption mechanism and multimode structural transformation property are revealed by both calculations and crystallography studies. This work demonstrates the feasibility of modulating flexibility for controlling gate‐opening effect, especially for some cases of significant aperture shrinkage after activation.

## Graphical abstract

Optimizing flexibility by encoding intraframework interactions relies on modulating different anion pillars. The shorter anion pillars play an important part in reducing the skeleton flexibility, which removes trace acetylene from acetylene/ethylene and acetylene/carbon dioxide mixtures simultaneously. It provides a switched approach to achieve the molecular sieving effect without precisely controlling aperture size but resorts to highly sensitive guest‐dependent threshold pressure control.

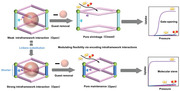

## 1 Introduction

An explosion in the research and development of new porous materials has been witnessed in the past two decades. For instance, the emergence of metal–organic frameworks (MOFs) thanks to their unique assembly, tailor‐made porosity, and surface properties which have great application potential in the area of gas separation and storage.^[^ ^1^ ^]^ In this context, porous MOFs for gas separation and purification are usually achieved by one or several of the following mechanisms, like thermodynamic equilibrium, kinetic, or even molecular sieving effect.^[^ ^2^ ^]^

Adsorption by means of molecular sieving with infinite selectivity is recognized as the most ideal separation approach. For highly similar molecules, such as alkynes/olefin and alkanes/olefin, it is particularly challenging to achieve sieving separation because of the difficulty of precise pore size control. Due to the virtually infinite number of inorganic and organic building blocks, potentially accessible MOFs with specific structure and function can be established.^[^ ^3^ ^]^ Rigid adsorbents identify different guest molecules by binding affinity discrepancy or size exclusion, while flexible MOFs can utilize multiple separation mechanisms, such as affinity and size/shape adaption, which are sometimes associated with a structural change promoted by guest–host accommodation.^[^ ^4^ ^]^ Combination of guest‐specific host–guest interactions with interior structural flexibility engineering will create unique functions.^[^ ^5^ ^]^ Therefore, it provides a switched approach to achieve the molecular sieving effect without precise controlling aperture size but rely on highly sensitive guest‐dependent threshold pressure. Several examples have certified that threshold pressure can be modulated by encoding the intraframework interactions (degree of flexibility).^[^ ^6^ ^]^ In principle, the flexibility of the framework is determined by the structure of the MOF and/or the external stimuli, such as pressure, temperature, and guests. Considering internal factors, the rigidity/flexibility of designable MOFs can be controlled by the proper selection of organic ligands and metal ions/clusters.^[^ ^7^ ^]^ The cumulative endeavor is being dedicated to establishing design principles to modulate the structural flexibility and transformations in flexible MOFs for specific applications, including linker or metal substitution and functionalization.

The separation of acetylene/ethylene (C_2_H_2_/C_2_H_4_) and acetylene/carbon dioxide (C_2_H_2_/CO_2_） by adsorption is recognized as an industrially promising process. A great number of MOFs have been examined as adsorbents for their separations, while most of them show coadsorption, and only a hand of materials can achieve C_2_H_2_/C_2_H_4_ sieving, such as UTSA‐200,^[^ ^8^ ^]^ UTSA‐300,^[^ ^9^ ^]^ NCU‐100,^[^ ^10^ ^]^ etc. Among, NCU‐100 had a low adsorption acetylene capacity of 0.73 mmol g^−1^ at 0.01 bar. Only two materials UTSA‐300 and CPL‐1‐NH_2_^[^ ^11^ ^]^ exhibit size sieving effect for C_2_H_2_/CO_2_ with both few acetylene uptakes at low pressure due to the flexibility of the structure, which are regarded as unsuitable for gas purification resulting from inevitably coadsorption of CO_2_. Ideal adsorption materials need both high adsorption capacity and selectivity. At present, the available MOFs or rational design strategies for high acetylene adsorption capacity at low pressure and almost exclusion of C_2_H_4_ and CO_2_ as well are still lacking.

**Figure 1:**
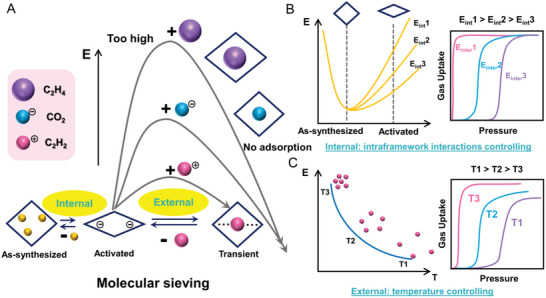
Encoding gate‐opening of flexible MOFs. A) The molecular sieving behaviors with structure flexibility toward size/affinity‐different guests, the positive and negative symbols only represent partial charge at one end of the molecules; control threshold pressure by B) internal different intraframework interactions as energy and adsorption profile shown and C) external different temperature tuning.

The adsorption behaviors within structural flexible MOF is different toward size/affinity‐different guests. Combination of internal intraframework interactions modulation and external different temperature tuning is an effective way to control threshold pressure to achieve trace impurity removal (**Figure** **1**). Herein, we reported an encoding gate‐opening of novel 2D layered flexible MOFs named Co(4‐DPDS)_2_MO_4_ (M = Cr, Mo, W) based on a linker substitution principle. Noteworthy, this kind of 2D MOFs started in the intralayer and interlayer pore‐opening configuration then local intralayer pore contracted after thermal activation without interlayer shrunken, followed by a transition to the pore reopening form for C_2_H_2_ molecules entering at relatively low pressures. Through judicious selection of shorter anions CrO_4_^2−^, enhanced multiple C—H···O hydrogen‐bonding interactions with organic linkers which weaken shrinkage of intralayer pores after activation. Therefore, the threshold pressure of C_2_H_2_ resulting from gate‐opening is precisely controlled so that Co(4‐DPDS)_2_CrO_4_ with less flexibility and proper opening size exhibits the C_2_H_2_ adsorption capacity is 0.60 mmol g^−1^ at 0.01 bar comparing to the C_2_H_2_ threshold pressure of 0.17 and 0.11 bar at 298 K on Co(4‐DPDS)_2_MoO_4_ and Co(4‐DPDS)_2_WO_4_, respectively. At the same time, the near exclusion of ethylene and carbon dioxide adsorption is retained, which strikes a notable balance between simultaneously high C_2_H_2_ working capacity and C_2_H_2_/C_2_H_4_ and C_2_H_2_/CO_2_ separation selectivity on Co(4‐DPDS)_2_CrO_4_ among the most state‐of‐the‐art MOFs. Direct crystallography results and DFT‐D calculations reveal prefer binding sites of C_2_H_2_ in three materials. Experimental results certify these materials display remarkable dynamic separation performance for C_2_H_2_/C_2_H_4_ and C_2_H_2_/CO_2_ mixtures as well as excellent stability and recyclability.

## 2 Results and Discussion

### 2.1 Structural Analysis and Flexible Encoding Mechanism

**Figure 2:**
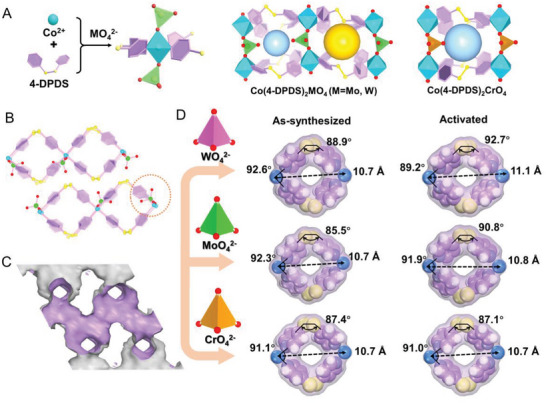
Schematic diagrams of structure. A) The coordination environment of Co (II), organic ligand 4‐DPDS and inorganic pillars MO_4_^2−^ and different cavities types in Co(4‐DPDS)_2_MO_4_. B) the 3D structure of Co(4‐DPDS)_2_MoO_4_ viewed along the c axis shows the 2D layered structure. C) Accessible Connolly surface of Co(4‐DPDS)_2_MoO_4_ determined by using a probe with the radius of 1.2 Å; D) comparison of the varying pore window of quasidiscrete cavities between Co(4‐DPDS)_2_MO_4_ and Co(4‐DPDS)_2_MO_4_a. H atoms were omitted for clarity in (A) and (B). Color scheme: Co, blue; O, red; pyridyl ring; purple; S, yellow; Cr, orange; Mo, green; W, rose.

The reaction of CoCl_2_·6H_2_O, 4,4‐dipyridyldisulfide (4‐DPDS) and K_2_CrO_4_, Na_2_MoO_4_, or Na_2_WO_4_, respectively, in an acetonitrile mixed aqueous solution and at room temperature yielded crystals of Co(4‐DPDS)_2_MO_4_ (M = Cr, Mo, W) (Figure S1, Supporting Information), which are isostructural with the monoclinic space group of P21/c in Co(4‐DPDS)_2_MoO_4_ and Co(4‐DPDS)_2_WO_4_, and C2/m in Co(4‐DPDS)_2_CrO_4_. In the as‐synthesized Co(4‐DPDS)_2_MO_4_, single‐crystal structure analysis revealed that each six‐coordinate Co (II) ion lies in an octahedral environment, which is formed by four nitrogen atoms from different 4‐DPDS ligands at the basal equatorial plane and two oxygen atoms from different MO_4_^2−^ pillars located at the axial positions, further creating an extending 2D sheet. A pseudocubic‐like cavity is formed by four adjacent Co (II) atoms via two pairs of double‐stranded 4‐DPDS bridges and two MO_4_^2−^ pillars. Among them, each 4‐DPDS group acts as a bidentate ligand whose the two pyridyl rings are almost perpendicular and exhibits a typical twisted conformation. Interestingly, there is a slight difference between Co(4‐DPDS)_2_MO_4_ (M = Mo, W) and Co(4‐DPDS)_2_CrO_4_. As seen in **Figure** **2**A, MoO_4_^2−^ and WO_4_^2−^ pillared in two alternate opposite directions, thus forming alternating cavities with different pore space. Nevertheless, every cavity possesses one oxygen atom showing homogeneous pore channel in Co(4‐DPDS)_2_CrO_4_ (Figure S2, Supporting Information). Besides, due to the chirality of 4‐DPDS,^[^ ^12^ ^]^ one pair of 4‐DPDS in Co(4‐DPDS)_2_CrO_4_ with opposite handedness was observed, which leads to a chair‐like centrosymmetric structure with a larger distinct window size. Such two adjacent 2D layers are further face‐to‐face stacking via numerous interlayer hydrogen bonds between frameworks and guest water molecules, cooperating together with *π*⋯*π* interactions among pyridyl rings and p⋯*π* interactions between S and pyridyl rings. Thus, three compounds all exhibit 2D layered structures with intralayer and interlayer space interlaced 3D channels.

**Figure 3:**
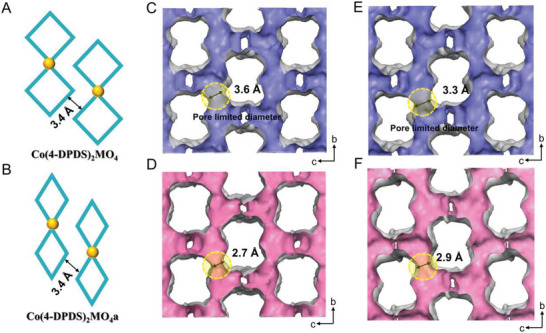
Comparison of crystal pore structures between Co(4‐DPDS)_2_MO_4_ and Co(4‐DPDS)_2_MO_4_a. A,B) Simplified diagram of shrinkage of intralayer pore windows after activated instead of interlayer distance, which is different from the layer to layer movement of conventional 2D MOFs; pore limited diameter in (C) Co(4‐DPDS)_2_MoO_4_ and (D) Co(4‐DPDS)_2_MoO_4_a; and in (E) Co(4‐DPDS)_2_WO_4_ and (F) Co(4‐DPDS)_2_WO_4_a.

Notably, after removal of water molecules, the pore window structure of Co(4‐DPDS)_2_MO_4_ changes significantly with distinct degrees (Figures 2D and **3**). In Co(4‐DPDS)_2_WO_4_, the C—S—S—C dihedral angle expands from 88.9° to 92.7°, the N—Co—N angle shrinks from 92.6^°^ to 89.2^°^, together with the Co···Co distance increases from 10.7 to 11.1 Å, which compresses inward from the square rhombus to the narrow long rhombus, resulting in the aperture window size as pore limited diameter (PLD) shrunk significantly from 3.3 to 2.9 Å. When the inorganic anion was replaced with MoO_4_^2−^, a similar contraction was observed. The movement degree of N—Co—N angle (0.4^°^ vs 3.4^°^) and Co···Co (0.1 Å vs 0.4 Å) distance was slightly smaller than Co(4‐DPDS)_2_WO_4_. And C—S—S—C bond (5.3^°^vs 3.8^°^) changes slightly more than Co(4‐DPDS)_2_WO_4_, which produced a smaller PLD in cavity (from 3.6 to 2.7 Å). In contrast, in Co(4‐DPDS)_2_CrO_4_, the dihedral angle of C—S—S—C has a slight change of 0.3^°^, but is significantly negligible compared to Co(4‐DPDS)_2_MoO_4_ (3.8^°^) and Co(4‐DPDS)_2_WO_4_ (5.3°). Therefore, the PLD in cavity of Co(4‐DPDS)_2_CrO_4_ is almost unchanged (3.8 Å). It hinted degree of structural transformation (not pore size) following the order: Co(4‐DPDS)_2_WO_4_ > Co(4‐DPDS)_2_MoO_4_ > Co(4‐DPDs)_2_CrO_4_. Simultaneously, owing to the tetrahedral anion pillars, there is little movement from layer to layer after activated and only the rotation of anions was observed in Co(4‐DPDS)_2_MoO_4_ and Co(4‐DPDS)_2_WO_4_. As a result, permanent interlayer channels with a slight enlargement in are demonstrated (Figures S3–S5, Supporting Information).

**Figure 4:**
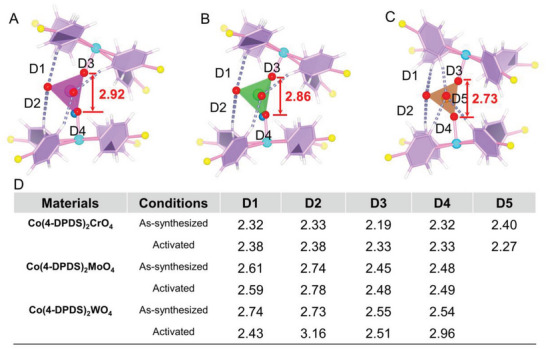
Intraframework interactions of Co(4‐DPDS)_2_MO_4_ and Co(4‐DPDS)_2_MO_4_a. A) Co(4‐DPDS)_2_WO_4_; B) Co(4‐DPDS)_2_MoO_4_; C) Co(4‐DPDS)_2_CrO_4_; D) the distance of existing intraframework hydrogen bond of as‐synthesized and after activated.

As envisaged, the relatively weak coordination ability of Co^2+^, which can be explained by the valence bond theory and crystal field theory, providing the preconditions for the obvious shrinkage of the pore window after removal of guest molecules. Since the uncoordinated O of the anion can interact with the H of pyridyl (D1–D5), the replacement of W^6+^ (1.41 Å) with Mo^6+^ (1.40 Å) and smaller Cr^6+^ (1.28 Å) is beneficial to the formation of multiple interactions in close proximity which enhance the stability of the internal structure (**Figure** **4**). Specifically, the anions O in Co(4‐DPDS)_2_MoO_4_ and Co(4‐DPDS)_2_WO_4_ interact with four pyridyl H simultaneously, while more hydrogen bonding was presented in Co(4‐DPDS)_2_CrO_4_, suggesting differences in binding energy of internal frameworks. The average hydrogen bonding of as‐synthesized Co(4‐DPDS)_2_CrO_4_ is 2.31 Å, which is much shorter than Co(4‐DPDS)_2_MoO_4_ (2.57 Å) and Co(4‐DPDS)_2_WO_4_ (2.64 Å). Accordingly, it can be expected that less structural flexibility in Co(4‐DPDS)_2_CrO_4_, also reflecting from the distance change between as‐synthesized phase and activated phase. Meanwhile, the average hydrogen bond distance after activation in Co(4‐DPDS)_2_MoO_4_ (2.585 Å) < Co(4‐DPDS)_2_WO_4_ (2.765 Å), indicating that the former might have a higher pore opening threshold pressure. It proves that the flexibility can be optimized by encoding intraframework interactions relying on modulating different anion pillars.

### 2.2 Structural Characterization

The as‐synthesized Co(4‐DPDS)_2_MO_4_ (M = Cr, Mo, W) frameworks were confirmed by powder X‐ray diffraction patterns (PXRD) (Figure S6, Supporting Information), which are in good agreement with the simulated patterns. After activation, Co(4‐DPDS)_2_MoO_4_ and Co(4‐DPDS)_2_WO_4_ obviously formed wider peaks at 13.7^°^, 15.7^°^, 20.0^°^, 24.2^°^, and 27.6^°^, while the crystal structure of Co(4‐DPDS)_2_CrO_4_ remains intact. It is proved that MoO_4_^2−^ and WO_4_^2−^ pillared structures are relatively more flexible, especially an obvious displacement of activated simulation pattern in Co(4‐DPDS)_2_WO_4_ was confirmed, further proving its greater flexibility. After the degassed material was soaked in pure water solution, the PXRD pattern was consistent with that of the newly synthesized material, which further verified that such a reversible structure change was caused by degassing. According to thermogravimetric analysis (TGA) (Figure S7, Supporting Information), the thermal stability of the three materials is up to 463 K. N_2_ at 77 K, Ar at 87 K, and CO_2_ at 195 K were used as probes intuitively determine the specific permanent porosity of the three materials, respectively (Figure S8, Supporting Information). On account of the large molecular kinetic diameters of N_2_ (3.64 Å) and Ar (3.60 Å), and their extremely weak affinity with the frameworks, no appreciable adsorption could be observed. In contrast, CO_2_ (3.3 Å) has a smaller molecule size and a stronger affinity toward the framework than N_2_ and Ar, showing a typical type I adsorption curve at very low pressures. The surface area calculated by Brunauer‐ Emmett‐Teller (BET) model is 92, 65, and 64 m^2^ g^−1^, respectively. Additionally, the porosity of three materials before and after activation is calculated. For as‐synthesized phases, Co(4‐DPDS)_2_CrO_4_, Co(4‐DPDS)_2_MoO_4_, and Co(4‐DPDS)_2_WO_4_ were calculated as 29.4%, 28.0%, and 28.3%, respectively, using Connolly probe molecules with radius of 1.65 Å. The calculated activated accessible voids are 29.4%, 27.4%, and 23.8%, respectively. Both experiment and calculation results indicated that the shorter CrO_4_^2−^ plays an important part in reducing the skeleton flexibility.

### 2.3 Single‐Component Gas Isotherms and Separation Performance

**Figure 5:**
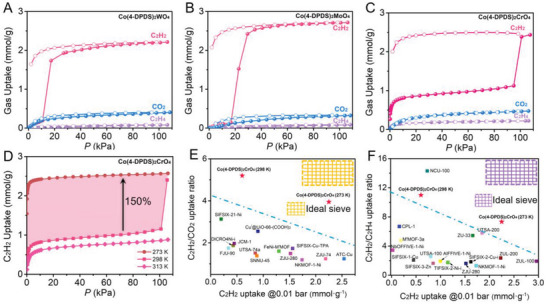
Gas adsorption isotherms and separation performance. Adsorption and desorption isotherms of C_2_H_2_ (pink), CO_2_ (blue), and C_2_H_4_ (purple) in A) Co(4‐DPDS)_2_WO_4_, B) Co(4‐DPDS)_2_MoO_4_, and C) Co(4‐DPDS)_2_CrO_4_ at 298 K. D) C_2_H_2_ adsorption isotherms in Co(4‐DPDS)_2_CrO_4_ at 273, 298, and 313 K in the region of 0–1 bar. Plots of the C_2_H_2_ uptake at 0.01 bar as a function of E) C_2_H_2_/CO_2_ uptake ratio and F) C_2_H_2_/C_2_H_4_ uptake ratio at 1.0 bar for Co(4‐DPDS)_2_CrO_4_ and the other benchmark materials for C_2_H_2_/CO_2_, C_2_H_2_/C_2_H_4_ separation at 298 K, respectively.

To confirm the feasibility of the strategy, the adsorption isotherms of C_2_H_2_, C_2_H_4_, and CO_2_ on Co(4‐DPDS)_2_MO_4_ (M = Cr, Mo, W) were collected at different temperatures from 273 to 313 K (**Figure** **5**; and Figure S9, Supporting Information). As predicted, the gate‐opening adsorption behavior was observed in Co(4‐DPDS)_2_MoO_4_ and Co(4‐DPDS)_2_WO_4_ at 298 K. Both materials demonstrated negligible acetylene capacities at low pressure, which abruptly increase when the pressures of C_2_H_2_ reach 0.17 and 0.11 bar, respectively. The adsorption capacities of C_2_H_2_ at 1 bar were 2.72 and 2.21 mmol g^−1^, indicating the opening of the pore. Conversely, due to the larger molecular kinetic diameter and the higher energy required, a total exclusion of ethylene (0.08 mmol g^−1^) was realized. Moreover, it revealed that Co(4‐DPDS)_2_MoO_4_ and Co(4‐DPDS)_2_WO_4_ exhibit the negligible CO_2_ uptake of 0.32 and 0.35 mmol g^−1^ at 298 K and 1 bar, respectively, and the adsorption trend at low pressure is consistent with the adsorption curve of C_2_H_2_, suggesting electrostatic repulsion resulted from opposite molecular quadrupole moment (C_2_H_2_, 20.5 × 10^−40^ C m^−2^; CO_2_, −13.4 × 10^−40^ C m^−2^) failed to open the closed‐pore structure. In contrast, Co(4‐DPDS)_2_CrO_4_ with less flexibility and proper opening size adsorbs C_2_H_2_ under the ultralow pressure and saturates rapidly, and the adsorption capacity of C_2_H_2_ at 0.01 bar is 0.60 mmol g^−1^. Interestingly, the adsorption capacity increased sharply to 2.43 mmol g^−1^ when it approaches 1 bar at 298 K, which may be caused by further opening of entrance pore windows and interlayer adsorption, and the repeatability of the data was confirmed (Figure S10, Supporting Information). Meanwhile, the adsorption capacity of C_2_H_4_ and CO_2_ were 0.22, 0.40 mmol g^−1^, respectively. In general, at lower temperatures, the thermal motion of gas is slower, and the molecular interaction between guest molecules and flexible MOFs is enhanced, so the gas molecules are more easily adsorbed on the flexible MOFs, thus reducing the “gate‐opening” pressure even achieving the removal of trace impurity. At 273 K, the threshold pressure of Co(4‐DPDS)_2_MoO_4_ and Co(4‐DPDS)_2_WO_4_ for C_2_H_2_ adsorption decreases to 0.06 and 0.05 bar, respectively. At the same time, the two‐step adsorption of C_2_H_2_ in Co(4‐DPDS)_2_CrO_4_ disappeared, and the adsorption capacity of C_2_H_2_ at 0.01 bar reached 2.25, and 2.57 mmol g^−1^ at 1 bar. The adsorption capacity of C_2_H_4_ in Co(4‐DPDS)_2_MoO_4_ and Co(4‐DPDS)_2_WO_4_ hardly increased, but in Co(4‐DPDS)_2_CrO_4_ slightly increased to 0.35 mmol g^−1^. It can be seen that the more flexible Co(4‐DPDS)_2_MoO_4_ and Co(4‐DPDS)_2_WO_4_ have high adsorption discrepancy for C_2_H_2_/C_2_H_4_ and C_2_H_2_/CO_2_, but may be deficient in removing trace acetylene. By regulating the internal (intraframework affinity) and external environment (temperature), Co(4‐DPDS)_2_CrO_4_ not only exhibits ultra‐low adsorption capacity for C_2_H_4_ and CO_2_, but also can capture a large amount of C_2_H_2_ at low pressure. These adsorption properties indicate that Co(4‐DPDS)_2_CrO_4_ has the greatest potential to remove trace C_2_H_2_ from C_2_H_2_/C_2_H_4_ and C_2_H_2_/CO_2_ mixtures simultaneously.

To better evaluate the separation ability of Co(4‐DPDS)_2_MO_4_ materials, especially Co(4‐DPDS)_2_CrO_4_, the ideal adsorbed solution theory (IAST) selectivity and adsorption capacity ratio of C_2_H_2_/C_2_H_4_ and C_2_H_2_/CO_2_ at different temperatures were calculated. By comparing the adsorption capacity of acetylene at 0.01 bar with the adsorption capacity ratio of C_2_H_2_/C_2_H_4_ and C_2_H_2_/CO_2_ at 1 bar, it can be found that Co(4‐DPDS)_2_CrO_4_ shows satisfactory results. As revealed in Figure 5E,F, the adsorption capacity ratio of C_2_H_2_/C_2_H_4_ on Co(4‐DPDS)_2_CrO_4_ is reaching 11 and 7.34 at 298 and 273 K, respectively, exceeding those of other top‐performing MOFs such as UTSA‐100 (0.8 mmol g^−1^, 2.57),^[^ ^13^ ^]^ UTSA‐200 (1.85 mmol g^−1^, 5.79),^[^ ^8^ ^]^ ZUL‐200 (2.23 mmol g^−1^, 2.36),^[^ ^14^ ^]^ NKMOF‐1‐Ni (1.73 mmol g^−1^, 1.29),^[^ ^15^ ^]^ SIFSIX‐2‐Cu‐i (1.62 mmol g^−1^, 1.84),^[^ ^16^ ^]^ ZJU‐280 (1.52 mmol g^−1^, 1.61)^[^ ^17^ ^]^ and other benchmark materials were second only to NCU‐100 (0.73 mmol g^−1^, 14.3).^[^ ^10^ ^]^ Co(4‐DPDS)_2_MoO_4_ and Co(4‐DPDS)_2_WO_4_ have higher adsorption capacity ratios of C_2_H_2_/C_2_H_4_, which are 34 and 27.6, respectively, at 298 K due to the lower adsorption capacity of C_2_H_4_. Additionally, at 273 and 298 K, the adsorption capacity ratio of C_2_H_2_/CO_2_ on Co(4‐DPDS)_2_CrO_4_ is 5.2 and 3.9, respectively, which is superior to most benchmark MOF materials recently reported, such as Cu^I^@UiO‐66‐(COOH)_2_ (2.55),^[^ ^18^ ^]^ ATC‐Cu (1.24),^[^ ^19^ ^]^ SIFSIX‐Cu‐TPA (1.73),^[^ ^20^ ^]^ SIFSIX‐21‐Ni (3.13),^[^ ^21^ ^]^ JCM‐1 (1.97),^[^ ^22^ ^]^ FJU‐90 (1.75),^[^ ^23^ ^]^ DICRO‐4‐Ni‐i (1.87).^[^ ^24^ ^]^ Meanwhile, the adsorption capacity ratio of C_2_H_2_/CO_2_ on Co(4‐DPDS)_2_MoO_4_ and Co(4‐DPDS)_2_WO_4_ is 8.5 and 5.5 at 298 K, respectively.

The calculated IAST selectivity of C_2_H_2_/C_2_H_4_ (1/99, *v*/*v*) and C_2_H_2_/CO_2_ (50/50, *v*/*v*) on Co(4‐DPDS)_2_CrO_4_ at 298 K were shown in Figures S11 and S12 (Supporting Information). The separation selectivity of C_2_H_2_/C_2_H_4_ (1:99, *v*:*v*) was analyzed to be 834 at 1 bar, 298 K, which also exceeded SIFSIX‐2‐Cu‐i (44.5),^[^ ^16^ ^]^ NKMOF‐1‐Ni (51.7),^[^ ^15^ ^]^ TIFSIX‐2‐Ni‐i (54.5),^[^ ^25^ ^]^ ZUL‐100 (175),^[^ ^14^ ^]^ ZU‐33 (1100)^[^ ^26^ ^]^ and other materials. What's more, it exhibits an ultrahigh C_2_H_2_/CO_2_ selectivity up to 302 at 1 bar (298 K). It presents a comprehensive comparison of Co(4‐DPDS)_2_CrO_4_ with the related MOFs in light of C_2_H_2_/C_2_H_4_ and C_2_H_2_/CO_2_ selectivities as concurrent objectives in Figure S13 (Supporting Information). Among these MOF, we found that Co(4‐DPDS)_2_CrO_4_ far exceeded previous ZJU‐280a (18.1, 44.5),^[^ ^17^ ^]^ NKMOF‐1‐Ni (22, 44),^[^ ^15^ ^]^ TIFSIX‐2‐Cu‐i (6.5, 55),^[^ ^25^ ^]^ JCM‐1 (13.7, 8.1),^[^ ^22^ ^]^ and MUF‐17 (6, 7.1),^[^ ^27^ ^]^ showing a rare combination of high C_2_H_2_/C_2_H_4_ and C_2_H_2_/CO_2_ selectivities. Evidently, Co(4‐DPDS)_2_CrO_4_ strikes a notable balance between simultaneously high C_2_H_2_ working capacity and C_2_H_2_/C_2_H_4_/CO_2_ separation selectivity among the reported MOFs. Such high affinity and specific capture for acetylene indicate the feasibility of the flexible strategy to regulate the strength of action in the skeleton.

Besides, the coverage‐dependent enthalpy of adsorption *Q*_st_ of C_2_H_2_ on Co(4‐DPDS)_2_MO_4_ (M = Cr, Mo, W) were calculated by Virial equation (Figure S14, Supporting Information). Among three materials, Co(4‐DPDS)_2_CrO_4_ and Co(4‐DPDS)_2_MoO_4_ possess higher *Q*_st_ values for C_2_H_2_,while the binding between C_2_H_2_ and Co(4‐DPDS)_2_WO_4_ is relatively weaker. It should be noted that the accuracy of Virial fitting for C_2_H_2_ on Co(4‐DPDS)_2_CrO_4_ is insufficient owing to the significant flexibility of the material, thus the calculated *Q*_st_ should be used for qualitative comparison only. Considering the negligible CO_2_ and C_2_H_4_ capacity of all three materials, the corresponding *Q*_st_ calculations were excluded.

### 2.4 Computational Simulation and Guest‐Loaded Single Crystal Structure

**Figure 6:**
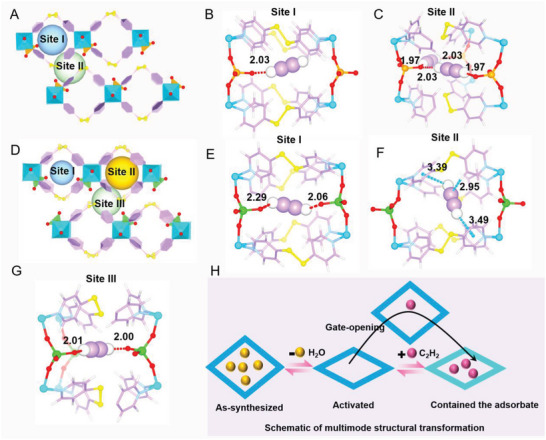
Schematic adsorption mechanism and calculated adsorption binding sites. A) Schematic diagram of two adsorption sites and DFT‐D calculated C_2_H_2_ adsorption binding B) site I and C) site II in Co(4‐DPDS)_2_CrO_4_. D) schematic diagram of three adsorption sites and DFT‐D calculated C_2_H_2_ adsorption binding E) site I, F) site II, and G) site III in Co(4‐DPDS)_2_MoO_4_. H) schematic adsorption mechanism with “gate‐opening” and pore is closed once the adsorbent entered.

To better understand the relationship between the selectivity and structure among three compounds, detailed first‐principles dispersion‐corrected density functional theory (DFT‐D) calculation was carried out to study the binding energies and optimal adsorption sites of acetylene. As shown in **Figure** **6**, for Co(4‐DPDS)_2_CrO_4_, there are two preferred binding sites (site I: intralayer channel and site II: interlayer channel). In site I, C_2_H_2_ mainly interacts with an uncoordinated O of CrO_4_^2‐^ atom in the form of C—H···O hydrogen bond at a distance of 2.03 Å, and the calculated binding energy is 36.7 kJ mol^−1^. In site II, the cooperative C—H···O hydrogen bond between the two ends of C_2_H_2_ and two adjacent uncoordinated O atoms occurs with a very short distance of 1.97 and 2.03 Å, respectively. The distance is much smaller than the sum of the van der Waals radii of H and O (2.6 Å), resulting in a high binding energy of 82.1 kJ mol^−1^. And Co(4‐DPDS)_2_MoO_4_ and Co(4‐DPDS)_2_WO_4_ showed three adsorption sites including two different intralayer sites and one interlayer site. As seen in site I of Co(4‐DPDS)_2_MoO_4_, the C_2_H_2_ molecule is located in the cage that contains two MoO_4_^2−^ ions in proximity interacting with the two terminal oxygen atoms with distance of 2.06, 2.29 Å, showing a higher binding energy (63.1 kJ mol^−1^) than Co(4‐DPDS)_2_CrO_4_ (site I) but lower than its interlayer adsorption binding energy. Additionally, each adsorbed C_2_H_2_ molecule in site II is surrounding by eight pyridyl rings and two pyridyl rings that form *δ*···*π* stacking interactions between the H(^*δ*+^) of C_2_H_2_ and the pyridyl *π* electrons, together with weak cooperative supramolecular interactions between C(^*δ*−^) of C_2_H_2_ and H(^*δ*+^) from —CH (C···H = 2.95 Å). Such interactions are weaker than the hydrogen bond, contributing to the C_2_H_2_ binding energy of 51.7 kJ mol^−1^. Similar to Co(4‐DPDS)_2_CrO_4_, a higher C_2_H_2_ binding energy of 71.9 kJ mol^−1^ in site III is revealed since it interacts with two O atoms with the shorter C—H···O hydrogen bond distance of 2.00 and 2.01 Å, respectively. As it is easier the shrinkage of the skeleton in Co(4‐DPDS)_2_WO_4_, the closer O···O distance at site I is not enough to contain C_2_H_2_ molecule, thus observed in a leaning manner which can also be proved by the closer interaction distance at site II. And the binding energies of C_2_H_2_ at site I, II, and III were 28.1, 49.3, and 73.2 kJ mol^−1^, respectively (Figure S15, Supporting Information).

To further visualize adsorption sites in three materials and verify the rationality of simulation, single crystal C_2_H_2_‐loaded X‐ray diffraction experiments were performed as illustrated in Figure S16 (Supporting Information). Clearly, the result revealed that C_2_H_2_ molecules can be simultaneously adsorbed in the intralayer and interlayer channels, which is consistent with the simulation results, but the actual sites are bound to locally optimal locations due to diffusion movement. In Co(4‐DPDS)_2_CrO_4_, only single adsorption in intralayer channel was observed. According to the previous single‐component adsorption behavior, this may be attributed to the high threshold pressure of opening the interlayer channels and insufficient adsorption pressure during the adsorption process. In addition, after loading C_2_H_2_ it can be seen that the XRD patterns at 13.7°, 15.7°, 20.0°, 24.2°, and 27.6° still form a wide peak, suggesting that the pores open and then close when the adsorbent passes through, interacting with the adsorbent in a closer way (Figure 6H; and Figure S17, Supporting Information).

### 2.5 Dynamic Column Breakthrough Studies

**Figure 7:**
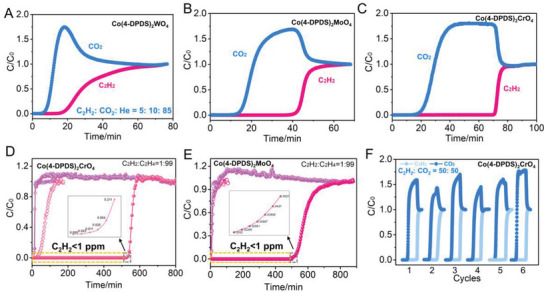
Experimental column breakthrough results. The curves for a C_2_H_2_/CO_2_/He (5/10/85, *v/v/v*) mixture in an absorber bed packed with A) Co(4‐DPDS)_2_WO_4_, B) Co(4‐DPDS)_2_MoO_4_, C) Co(4‐DPDS)_2_CrO_4_ at 298 K, respectively; comparison of experimental column breakthrough curves for a C_2_H_2_/C_2_H_4_ (1/99, *v*/*v*) mixture of D) Co(4‐DPDS)_2_CrO_4_ and E) Co(4‐DPDS)_2_MoO_4_ at 273 and 298 K. F) The cycling breakthrough test of C_2_H_2_/CO_2_ (50/50, *v*/*v*) mixture with a total flow of 0.7 mL min^−1^ in an absorber bed packed with Co(4‐DPDS)_2_CrO_4_ at 298 K and 1 bar.

In order to investigate the practical separation performance of Co(4‐DPDS)_2_MO_4_ (M = Cr, Mo, W) for C_2_H_2_/C_2_H_4_ and C_2_H_2_/CO_2_, breakthrough experiments were carried out with a 1/99, 50/50 C_2_H_2_/C_2_H_4_ mixtures as well as C_2_H_2_/CO_2_ (50/50, *v*/*v*) and C_2_H_2_/CO_2_/He (5/10/85, *v*/*v*/*v*) mixtures through packed columns. As expected in Figure S18 (Supporting Information), Co(4‐DPDS)_2_CrO_4_ achieved complete separation of 1/99 C_2_H_2_/C_2_H_4_ at 298 K, with ethylene soon eluting and rapidly reaching the inlet concentration, while acetylene was completely absorbed into the column and slowly breakthrough until 25 min later. Conversely, a simultaneous breakthrough of C_2_H_2_ and C_2_H_4_ occurs of 1/99 C_2_H_2_/C_2_H_4_ gas mixture at once in Co(4‐DPDS)_2_MoO_4_ and Co(4‐DPDS)_2_WO_4_ at 298 K, making them unsuitable for gas purification in which trace impurities from gas mixtures need to be efficiently removed. From the 50/50 mixture separation results, the three materials all achieve good separation of C_2_H_2_/C_2_H_4_. Especially in Co(4‐DPDS)_2_MoO_4_ and Co(4‐DPDS)_2_WO_4_, due to the increase of acetylene concentration to get pore opening, it will inevitably cause ethylene coadsorption. As significantly increased acetylene adsorption capacity and decreased threshold pressure at low temperature, 1/99 C_2_H_2_/C_2_H_4_ separation experiment of Co(4‐DPDS)_2_CrO_4_ and Co(4‐DPDS)_2_MoO_4_ were carried out at 273 K. Although the actual flow rate decreased slightly, it was found that the separation performance was greatly improved both in Co(4‐DPDS)_2_CrO_4_ and Co(4‐DPDS)_2_MoO_4_. The retention time of acetylene in Co(4‐DPDS)_2_CrO_4_ was extended from the previous 25 min to more than 500 min with the rapid breakthrough of acetylene, and the ethylene yield (99.999%) increased from 2.06 to 78.5 mmol g^−1^. At 273 K, a very impressive separation effect in Co(4‐DPDS)_2_MoO_4_ achieved as well, and the yield of 99.999% ethylene is 34.2 mmol g^−1^, suggesting their high potential as a C_2_H_2_/C_2_H_4_ separation material and temperature has a great influence on the adsorption of flexible materials (**Figure** **7**).

Among the three, Co(4‐DPDS)_2_CrO_4_ showed the best separation performance with the shorter mass transfer zone at low concentration C_2_H_2_/CO_2_ separation (Figure 7A–C). When the equimolar C_2_H_2_/CO_2_ entered the packed column at 298 K, the phenomenon of leakage appeared in Co(4‐DPDS)_2_MoO_4_ and Co(4‐DPDS)_2_WO_4_, and the degree of leakage was consistent with the threshold pressure of acetylene. At 273 K, the leakage of Co(4‐DPDS)_2_MoO_4_ almost disappeared, and acetylene began to leak after remaining in Co(4‐DPDS)_2_WO_4_ for quite a long time (Figure S19, Supporting Information). According to the dynamic adsorption capacity ratio at 273 K, the separation factors of Co(4‐DPDS)_2_WO_4_ (7.2) > Co(4‐DPDS)_2_MoO_4_ (5.1) > Co(4‐DPDS)_2_CrO_4_ (4.7), owing to the lower CO_2_ adsorption capacity of the former. Compared with other materials, the calculated separation factor of the material is higher than that of JCM‐1 (4.4),^[^ ^22^ ^]^ ZJU‐74a (4.3),^[^ ^28^ ^]^ CAU‐10‐H (3.4),^[^ ^29^ ^]^ Cu^I^@UiO‐66‐(COOH)_2_ (3.4),^[^ ^18^ ^]^ SNNU‐45 (2.9)^[^ ^30^ ^]^ et al. The separation factor of Co(4‐DPDS)_2_CrO_4_ (3.6) at 298 K was slightly lower than that of JCM‐1 and ZJU‐74a (Figure S20, Supporting Information).

### 2.6 Stability and Recyclability

For a real‐world application, the candidate adsorbents should have excellent thermal stability, water stability, and chemical stability. The PXRD pattern proves that three samples retain good crystallinity even after being exposed to air or immersed in water for one month, indicating its excellent stability. Additionally, the chemical stability of Co(4‐DPDS)_2_MO_4_ (M = Cr, Mo, W) was assessed by treating samples in a strong acid (pH = 1) and base (pH = 10) solution (Figure S21, Supporting Information). It can be seen from PXRD that the crystal integrity can be maintained even after soaking in strong acid and base as long as a month, indicating that there is no phase transformation or structural collapse. The C_2_H_2_ capacities of samples after the stability tests also keep nearly unchanged (Figure S22, Supporting Information), but slightly degrade after treated with acid, which further manifested the excellent stability of all three materials. Since Co(4‐DPDS)_2_CrO_4_ showed the best separation potential in ethylene purification and C_2_H_2_/CO_2_ separation, the cyclic and impact of water vapor breakthrough experiments were carried out to evaluate the recovery and separation ability of C_2_H_2_/CO_2_ separation as a representative under humid environment (Figure S23, Supporting Information). Figure 7F embodied no noticeable decrease of breakthrough performance of C_2_H_2_ within six cycles revealing the excellent recyclability of this material for the C_2_H_2_/CO_2_ separation. The breakthrough experiment at 100% humidity proves that the separation ability is not disturbed by water vapor.

## 3 Conclusion

In summary, fine‐tuning of gate‐opening effect through intraframework interactions in a series of novel 2D layered MOFs has provided insight into interplay of intrinsic structural flexibility and separation performance of C_2_H_2_/C_2_H_4_ and C_2_H_2_/CO_2_. The anion pillars substitution strategy certifies the shortest CrO_4_^2−^ can offer multiple‐point interactions of intraframework in close proximity so that negligible pore windows contraction is realized after activation. As a successful demonstration of this strategy, Co(4‐DPDS)_2_CrO_4_ exhibits high C_2_H_2_ uptake at ultralow pressure as well as superior C_2_H_2_/C_2_H_4_ and C_2_H_2_/CO_2_ separation performance simultaneously with nearly molecular sieving, which strikes a good balance among working capacity, separation selectivity and trace impurity removal of flexible MOFs. Both of the theoretical calculations and direct C_2_H_2_‐loaded experimental results not only confirmed the angular inorganic anions provides polar sites for specific C_2_H_2_ recognition and pore confinement within the cavity surrounding by eight pyridyl rings, but also an exceptional multimode structural transformation ability is revealed. This work provides an effective guidance on tuning intrinsic flexibility on adsorption properties via programming intraframework interactions according to gas‐sorbent interactions discrepancy of targets within flexible MOFs.

## 4 Experimental Section

The experimental details are listed in the Supporting Information.

CCDC: 2 160 490 (Co(4‐DPDS)_2_CrO_4_), 2 160 489 (Co(4‐DPDS)_2_CrO_4_a), 2 160 491 (Co(4‐DPDS)_2_MoO_4_), 2 160 496 (Co(4‐DPDS)_2_MoO_4_a), 2 160 495 (Co(4‐DPDS)_2_WO_4_), 2 160 492 (Co(4‐DPDS)_2_WO_4_a), 2 160 493 (Co(4‐DPDS)_2_CrO_4_⊃C_2_H_2_), 2 160 494 (Co(4‐DPDS)_2_MoO_4_⊃C_2_H_2_), and 2 160 497 (Co(4‐DPDS)_2_WO_4_⊃C_2_H_2_) contains the supplementary crystallographic data for this paper. These data can be obtained free of charge from The Cambridge Crystallographic Data Centre via www.ccdc.cam.ac.uk/data_request/cif.

## Conflict of Interest

The authors declare no conflict of interest.

## Supporting information

Supporting Information

## Data Availability

The data that support the findings of this study are available from the corresponding author upon reasonable request.
