# Supplementary material for: Strengthening Intraframework Interaction within Flexible MOFs Demonstrates Simultaneous Sieving Acetylene from Ethylene and Carbon Dioxide
Source: Adv Sci (Weinh). 2023 Jan 26;10(9):2207127. doi: 10.1002/advs.202207127 (PMC10037686; doi:10.1002/advs.202207127)
Supplement: Supplementary file 1 — Supporting Information [file ADVS-10-2207127-s001.pdf]

ADVANCED SCIENCE
Open Access

Supporting Information

for *Adv. Sci.*, DOI 10.1002/advs.202207127

Strengthening Intraframework Interaction within Flexible MOFs Demonstrates Simultaneous Sieving Acetylene from Ethylene and Carbon Dioxide

*Fang Zheng, Rundao Chen, Ying Liu, Qiwei Yang, Zhiguo Zhang, Yiwen Yang, Qilong Ren and Zongbi Bao**

Supplementary Information

Strengthening intra-framework interaction within flexible MOFs demonstrates simultaneous sieving acetylene from ethylene and carbon dioxide

Fang Zheng, Rundao Chen, Ying Liu, Qiwei Yang, Zhiguo Zhang, Yiwen Yang, Qilong Ren, Zongbi Bao *

General Information and Procedures

Materials

Cobalt (II) chloride hexahydrate ($CoCl_2·6H_2O$, 98%) was obtained from Sigma-Aldrich, and 4,4'-dipyridyldisulfide (4-DPDS, 98%), sodium molybdenum oxide, anhydrous (Na_2MoO_4, 99%) and sodium tungstate, dihydrate ($Na_2WO_4·2H_2O$, 99.5%) were purchased from Aladdin, and potassium chromate (K_2CrO_4, >99.5%) was purchased from Guangdong Guanghua Chemical Factory Co.,Ltd. All other chemicals (AR grade) were used as received from commercial suppliers without further purification. Ultrahigh purity grade He (99.999%), CO_2 (99.999%), C_2H_2 (99.999%), N_2 (99.999%), and mixed gases of C_2H_2/C_2H_4 = 1:99 (*v/v*), C_2H_2/C_2H_4 = 50/50 (*v/v*), C_2H_2/CO_2 = 50/50 (*v/v*) and C_2H_2/CO_2/He = 5/10/85 (*v/v/v*) from Jingong Co., Ltd. (China) and used for all measurements.

Synthesis of Co(4-DPDS)$_2$MO$_4$ (M = Cr, Mo, W) Materials

An acetonitrile solution (20 mL) containing 4-DPDS (220 mg,1 mmol) was added to the aqueous solution (20 mL) containing $CoCl_2·6H_2O$ (119 mg, 0.5 mmol) and K_2CrO_4 (97 mg, 0.5 mmol) at room temperature. The obtained Co(4-DPDS)$_2$CrO$_4$ mixture was stirred overnight and the precipitate was then filtered and washed with water and acetonitrile, followed by drying for several hours at room temperature. Synthesis of Co(4-DPDS)$_2$MoO$_4$ were prepared in the same way as Co(4-DPDS)$_2$CrO$_4$ except that

Na_2MoO_4 (103 mg, 0.5 mmol) was used instead of K_2CrO_4. Synthesis of Co(4-DPDS)$_2$WO$_4$ were prepared in the same way as Co(4-DPDS)$_2$CrO$_4$ except that $Na_2WO_4 \cdot 2H_2O$ (119 mg, 0.5 mmol) was used instead of K_2CrO_4.

Synthesis for X ray single crystal diffraction analysis

An aqueous solution (10 mL) of $CoCl_2 \cdot 6H_2O$ (23.7 mg, 0.1 mmol) was mixed with an aqueous solution (10 mL) of K_2CrO_4 (19.7 mg, 0.1 mmol) in a long thin test tube, the resulting solution was carefully layered under 2 mL of acetonitrile and water (*v*/*v* = 1:2), then layered under 4-DPDS (44.0 mg, 0.2 mmol) in 10 mL of acetonitrile and water (*v*/*v* = 2:1). The tube was sealed and left undisturbed at room temperature. After a week red-brown block-shaped crystal of Co(4-DPDS)$_2$CrO$_4$ were isolated. Crystals of Co(4-DPDS)$_2$MoO$_4$ were prepared in the same way as Co(4-DPDS)$_2$CrO$_4$ except that Na_2MoO_4 (20.6 mg, 0.1 mmol) was used instead of K_2CrO_4. After a week, the pink block-shaped crystals were harvested. Crystals of Co(4-DPDS)$_2$WO$_4$ were prepared in the same way as Co(4-DPDS)$_2$CrO$_4$ except that $Na_2WO_4 \cdot 2H_2O$ (23.8 mg, 0.1 mmol) was used instead of K_2CrO_4. After a week, the pink block-shaped crystals were harvested.

C_2H_2-loaded analysis

Firstly, the single crystals were loaded into the sample tube for adsorption test after degas, then, C_2H_2 gas was backfilled. After single crystals were sealed by epoxy resin, they were taken out and tested on a Bruker APEX-II CCD diffractometer.

Characterization

Power X-ray diffraction (PXRD) analysis of powder samples was carried out on an X'Pert diffractometer (PANalytical Corp., Netherlands) using Cu Kα (λ = 0.1543 nm) radiation at 40 kV/40 mA from 5°to 45° (2θ angle range) in a step of 0.02° per second. The single crystal X-ray diffraction of the as-synthesized and activated samples were performed at 193 K using a Bruker D8 VENTURE PHOTON II system equipped with a Liquid Metal source MetalJet (Ga Kα, λ = 1.34139 Å) and a Helios Multilayer Optic monochromator. Using Olex2, the structure was solved with the SHELXT

structure solution program using Intrinsic Phasing and refined with the SHELXL refinement package using Least Squares minimization. Detailed crystallographic data are summarized in Table S1. The thermal gravimetric analysis (TGA) was performed in TA-Q500 (TA Instruments) with heating rate of 10 °C/min under N_2 circumstances from 50 to 800 °C. Adsorption-desorption isotherms of N_2 at 77 K, Ar at 87 K and CO_2 at 195 K were obtained on 3Flex-3MP adsorption apparatus (Micrometrics).

PXRD measurements for stability test of material

As-synthesized samples after being washed with deionized water, about 0.2 g for each batch, were immersed in 10 mL of aqueous solution of pH = 1 (HCl), and pH = 10 (NaOH) at room temperature for 24 h, a week and a month, respectively. The treated samples were washed with deionized water for several times and dried at room temperature before PXRD measurements.

Gas adsorption measurements

Single-component isotherms of C_2H_2, C_2H_4 and CO_2 were measured up to 1 bar at 313, 298 and 273 K, on Micrometrics ASAP 2460 adsorption apparatus, respectively. The initial outgassing process was carried out under high vacuum (< 4 mmHg) at 100 °C for 24 h before adsorption measurements and about 120 mg sample was used for each gas adsorption measurement. The free space of the system was determined by using helium gas.

Calculation of adsorption selectivity

The dual-site Langmuir-Freundlich (DSLF) model is used to describe the gas adsorption isotherms.[1] The model is well-defined as:

$$q = q_{A,sat}\frac{b_A P^{n_A}}{1+b_A P^{n_A}} + q_{B,sat}\frac{b_B P^{n_B}}{1+b_B P^{n_B}} \quad (1)$$

where $q_{\mathrm{A,sat}}$ and $q_{\mathrm{B,sat}}$ are the saturated capacities of site A and site B, b_{A} and b_{B} are the affinity coefficients to the sites A and B, P is the pressure of the bulk gas at equilibrium with the adsorbed phase, q is the gas uptake amount of an adsorbent, and n_{A} and n_{B} represent the deviations from an ideal homogeneous surface. Prediction of the selectivity was established using ideal adsorption solution theory (IAST) [1]

combined with DSLF for binary gas on porous materials. Herein, the adsorption selectivity is defined by:

$$S = \frac{q_1/q_2}{p_1/p_2} \tag{2}$$

q_1, and q_2 are the molar loadings in the adsorbed phase in equilibrium with the bulk gas phase with partial pressures p_1 and p_2. The fitting parameters of the DSLF equation for C_2H_2, C_2H_4, and CO_2 at 298 K, respectively are presented in Supplementary Table 5.

Calculation of adsorption enthalpy Q_{st}

The virial equation is used to fit the single-component isotherms and calculate the Q_{st}, defined as:

$$\ln(P) = \ln(N) + \frac{1}{T}\sum_{i=0}^{m} a_i \times N^i + \sum_{j=0}^{n} b_j \times N^j \tag{3}$$

$$Q_{ST} = -R \times \sum_{i=0}^{m} a_i \times N^i \tag{4}$$

A virial-type expression of the above form was used to fit the combined isotherm data at 298 and 313 K. Where P (mmHg) is the pressure, N (mg/g) is the adsorbed amount of adsorbed gas, T (K) is the temperature, a_i and b_j are virial coefficients, m and n are the numbers of coefficients used to describe the isotherms. Q_{st} (kJ/mol) is the coverage-dependent enthalpy of adsorption.

Breakthrough experiments

Breakthrough tests were conducted in a stainless-steel column (50 mm x 4.6 mm ID) manually packed according to different particle size and density of the sample powder. The weight packed in the column was: 0.3786 g Co(4-DPDS)$_2$CrO$_4$, 0.4643 g Co(4-DPDS)$_2$MoO$_4$, 0.4257g Co(4-DPDS)$_2$WO$_4$ samples, respectively. The GC2010 Pro gas chromatography was used for continuous sampling analysis of the outlet gas passing through FID detector for C_2H_2/C_2H_4 (50:50, *v/v*) and C_2H_2/C_2H_4 (1:99, *v/v*) of a flow rate of 2 mL/min at 273 and 298 K. And under the same conditions, the binary mixture of C_2H_2/CO_2 (50:50, *v/v*) or ternary mixture of C_2H_2/CO_2/He (5:10:85, *v/v/v*) were introduced with a flow rate of 1 mL/min at 273 or 298 K, respectively. The outlet gas passing through the column was analyzed using a Hiden HPR-20 EGA mass

spectrometer for continuous sampling gas analysis. After the breakthrough tests, the column was regenerated with a He gas flow of 14.0 mL/min at room temperature for 48 h. Before breakthrough tests, samples were initially activated as mentioned above for 24 h under ultrahigh vacuum. The breakthrough experiments using bubbler were further carried out to compare the effect of moisture on dynamic. The breakthrough experiments were conducted under a mixture of C_2H_2/CO_2 (50:50, *v*/*v*) with a total flow of 5 mL/min at 298 K.

Separation factor and dynamic capacity

The dynamic capture capacity of the gas is calculated as:

$$n_{adsi} = FC_i t_i \quad (5)$$

Where n_{adsi} is the adsorption capacity of the gas i, *F* is the total molar flow, C_i is the concentration of the gas i entering the column and the time corresponding to the gas i, which is estimated from the breakthrough profile. The selectivity was then calculated according to the equation:

$$S = \frac{x_a/x_b}{y_a/y_b} \quad (6)$$

Where x_a and x_b are the mole fractions of the gases *a* and *b* in the adsorbed phase and y_a and y_b are the mole fractions of the gases *a* and *b* in the bulk phase.

Density-functional theory calculations

First principles density-functional theory (DFT) calculations were performed using the Materials Studio's CASTEP code[2]. A semi-empirical addition of dispersive forces to conventional DFT was included in the calculation to account for van der Waals interactions. We use Vanderbilt-type ultrasoft pseudopotentials and generalized gradient approximation (GGA) with Perdew-Burke-Ernzerhof (PBE) exchange correlation. A cut-off energy of 544 eV and 1 × 2 × 1 *k*-point mesh (generated using the Monkhorst–Pack scheme) were found to be enough for the total energy to converge within 0.01 meV·atom^{-1}. The structure of compounds (as-synthesized) was first optimized. The optimized structures are in good agreement with the experimentally

determined crystal structures of the coordination networks. C_2H_2 molecule was then separately introduced to various locations of the channel pore, followed by a full structural relaxed. To obtain the gas binding energy, a gas molecule placed in a supercell with the same cell dimensions was relaxed as a reference. The static binding energy (at T = 0 K) was then calculated using:

$$E_B=E_{(MOF+gas\_molecule)} - E_{(MOF)} - E_{(gas\_molecule)} \quad (7)$$

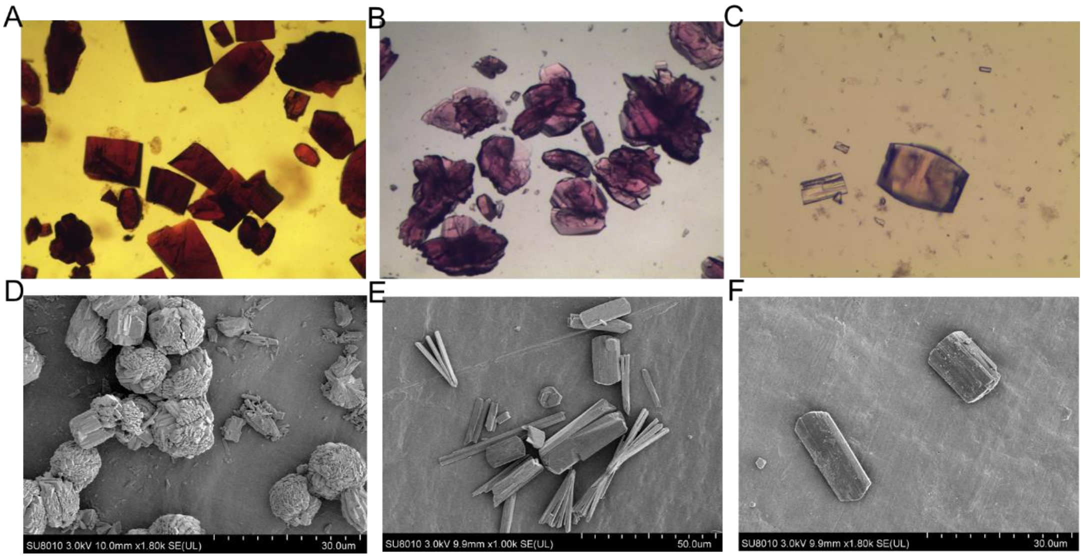

Figure S1. The microscope images of as-synthesized crystal samples and SEM images of as-synthesized powder samples: (A) (D) Co(4-DPDS)$_2$CrO$_4$; (B) (E) Co(4-DPDS)$_2$MoO$_4$; (C) (F) Co(4-DPDS)$_2$WO$_4$.

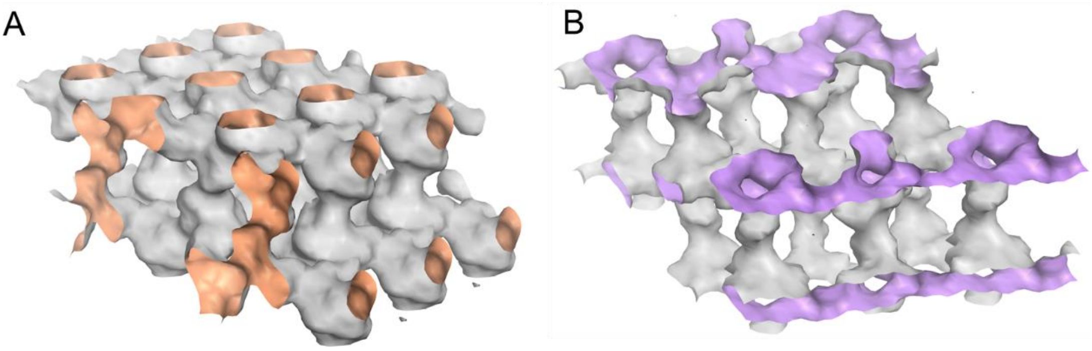

Figure S2. Discrete channels of different anion pillared materials. (A) Co(4-DPDS)$_2$CrO$_4$ has homogeneous channels; (B) Co(4-DPDS)$_2$MoO$_4$ and Co(4-DPDS)$_2$WO$_4$ have two different sizes of pore structures.

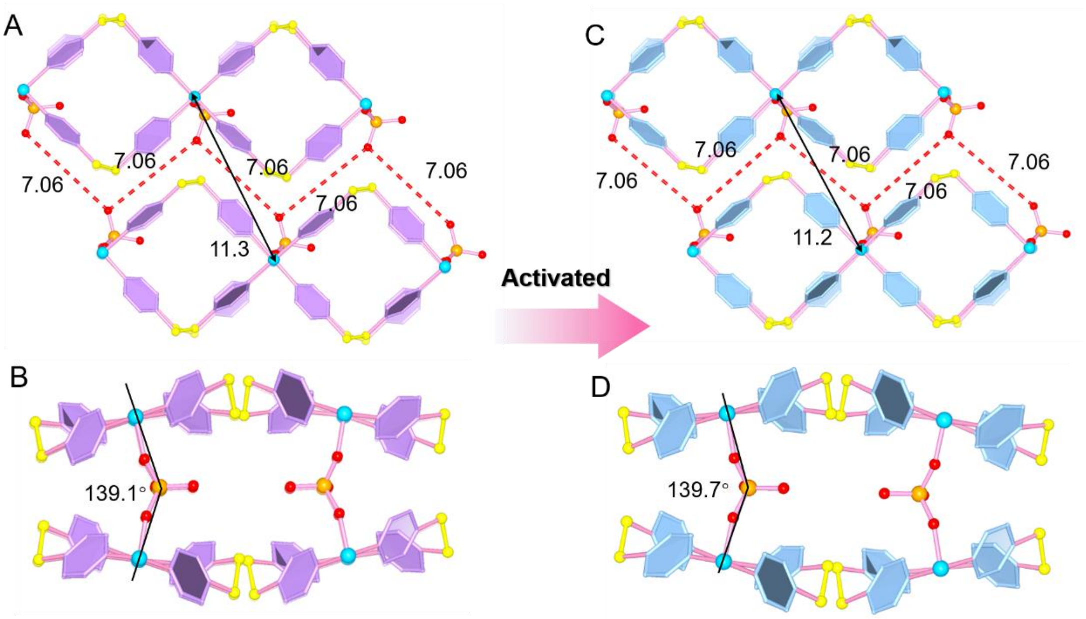

Figure S3. The as-synthesized (A) (B) and activated (C) (D) crystal structure of Co(4-DPDS)$_2$CrO$_4$. The pore structure and size remained almost unchanged after activation.

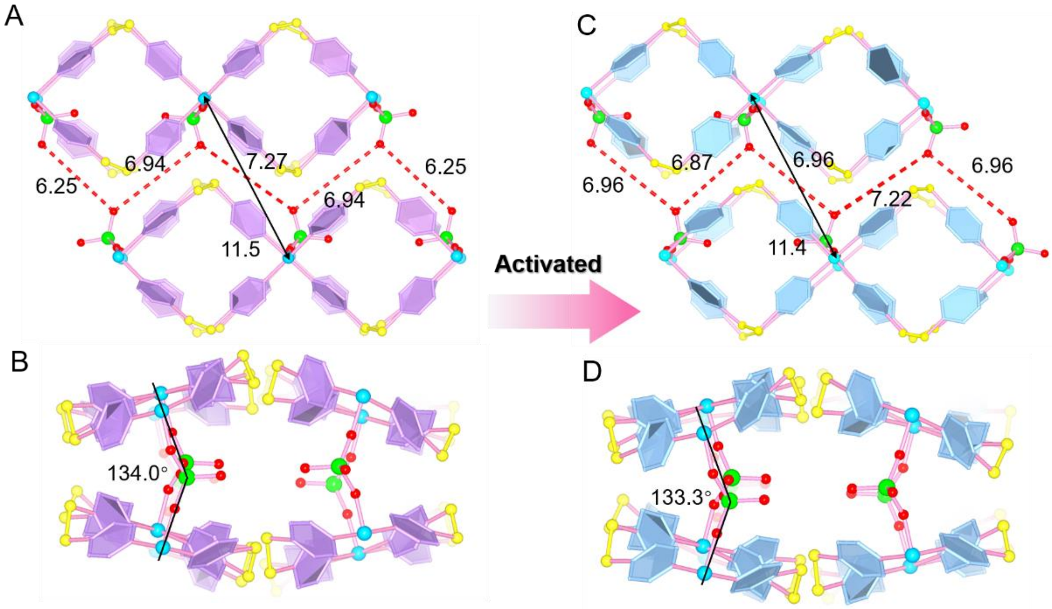

Figure S4. The as-synthesized (A) (B) and activated (C) (D) crystal structure of Co(4-DPDS)$_2$MoO$_4$. The activated crystal structure becomes slightly disordered.

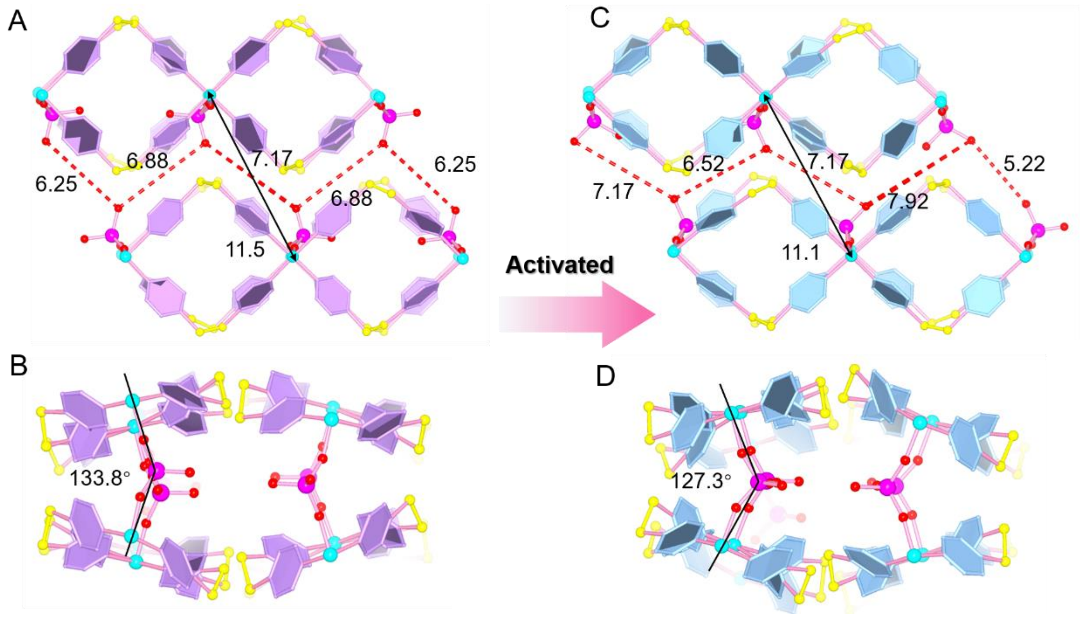

Figure S5. The as-synthesized (A) (B) and activated (C) (D) crystal structure of Co(4-DPDS)$_2$WO$_4$. The activated crystal structure becomes slightly disordered.

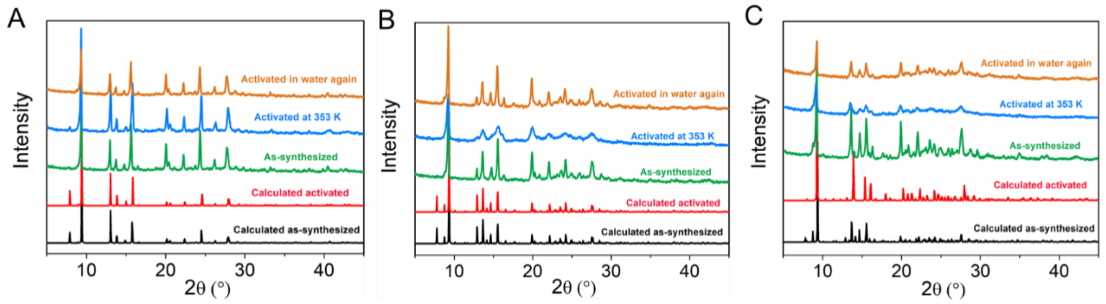

Figure S6. PXRD patterns of (A) Co(4-DPDS)$_2$CrO$_4$; (B) Co(4-DPDS)$_2$MoO$_4$; (C) Co(4-DPDS)$_2$WO$_4$.

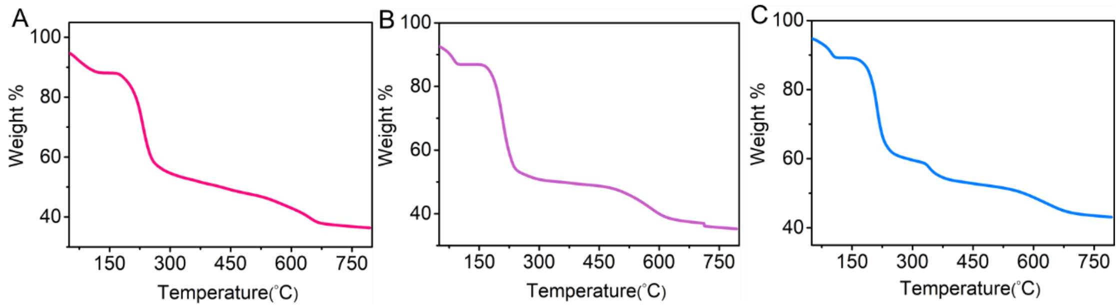

Figure S7. TGA curves of (A) Co(4-DPDS)$_2$CrO$_4$; (B) Co(4-DPDS)$_2$MoO$_4$; (C) Co(4-DPDS)$_2$WO$_4$.

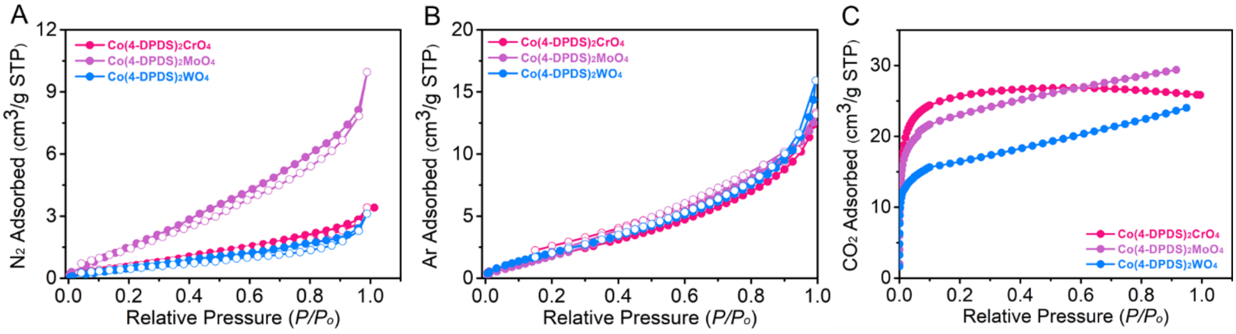

Figure S8. (A) The N_2 adsorption isotherms at 77 K; (B) the Ar adsorption isotherms at 87 K; (C) the CO_2 adsorption isotherms at 195 K of as-synthesized samples.

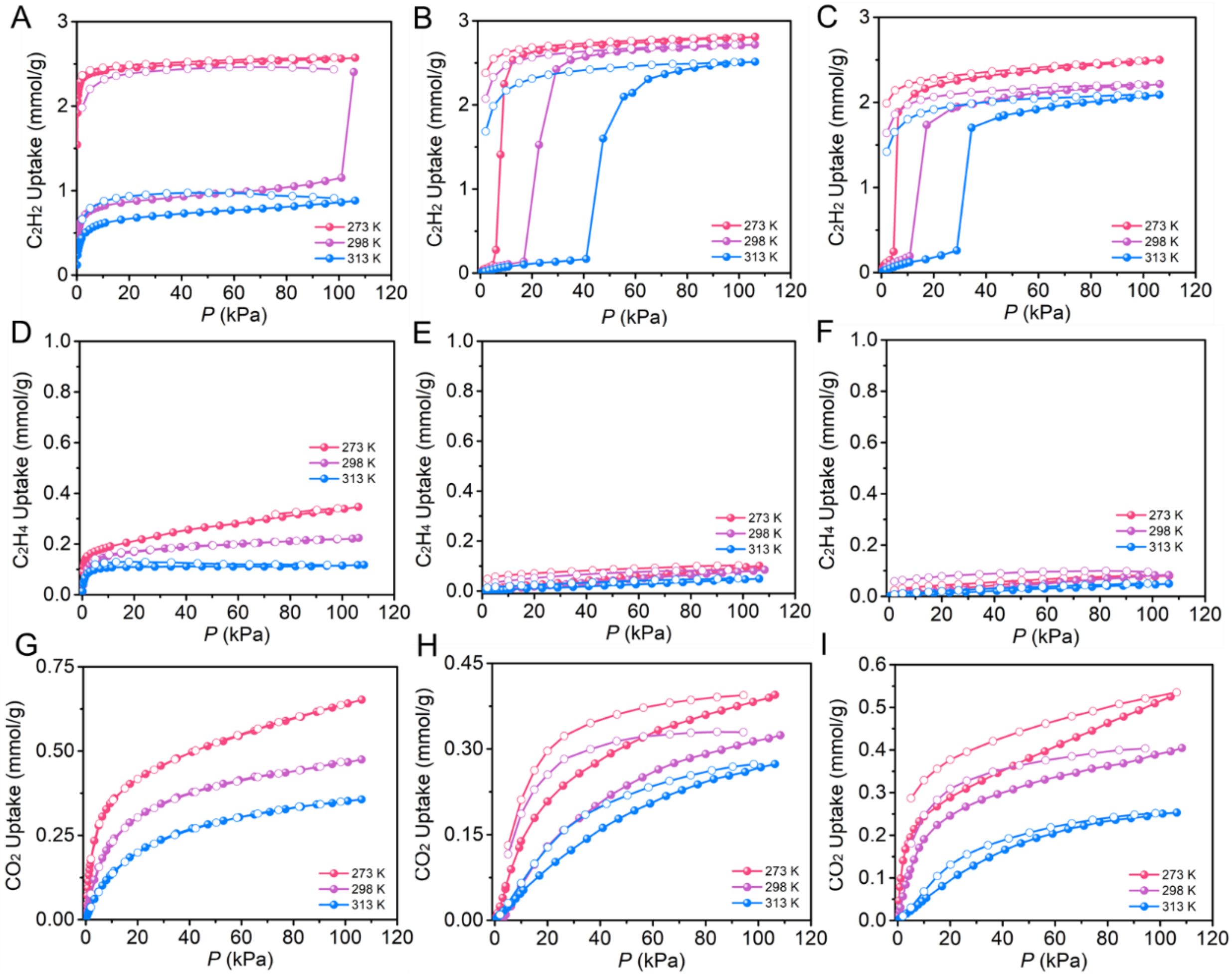

Figure S9. Single component adsorption isotherms of acetylene (A-C), ethylene (D-F) and carbon dioxide (G-I) at 0~1 bar at 273, 298 and 313 K, respectively. (A) (D) (G) Co(4-DPDS)$_2$CrO$_4$, (B) (E) (H) Co(4-DPDS)$_2$MoO$_4$, (C) (F) (I) Co(4-DPDS)$_2$WO$_4$.

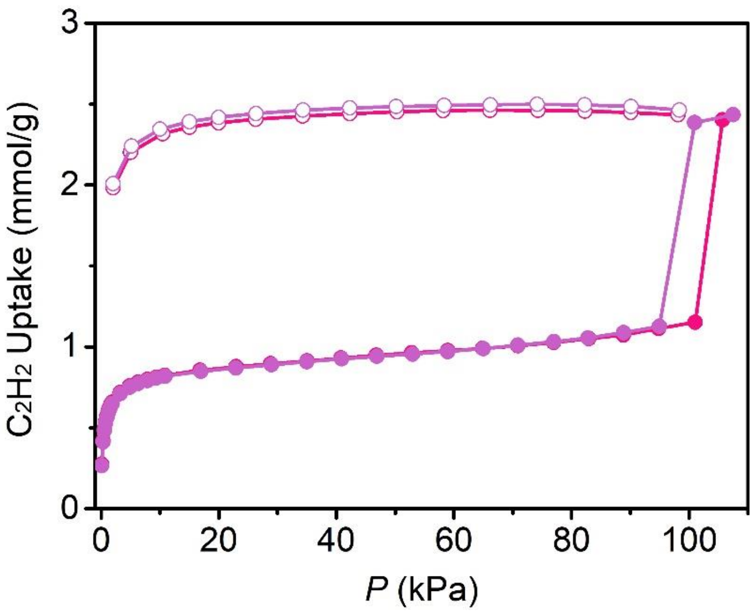

Figure S10. Repeated single component adsorption isotherms of acetylene on Co(4-DPDS)$_2$CrO$_4$ at 298 K.

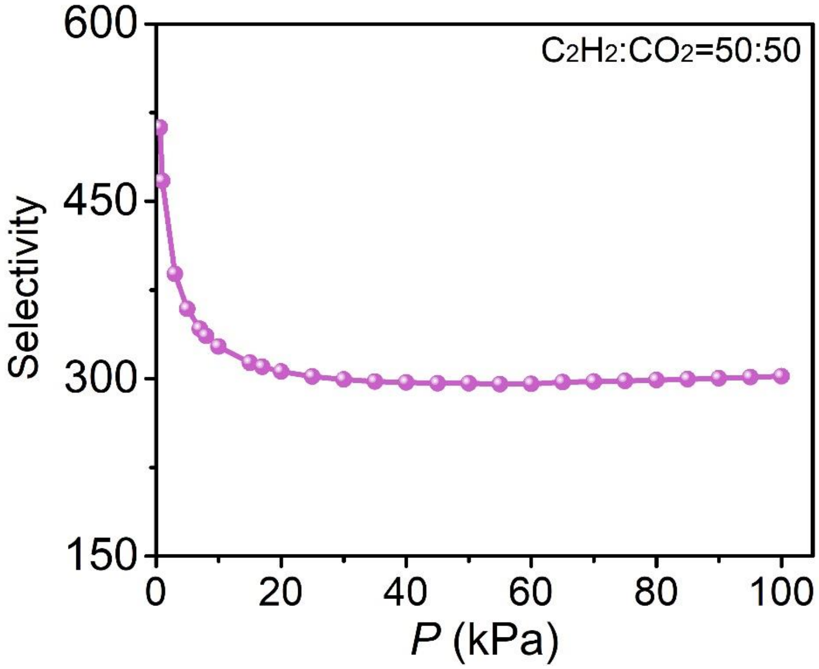

Figure S11. IAST selectivity of Co(4-DPDS)$_2$CrO$_4$ for a 50/50 C_2H_2/CO_2 mixture.

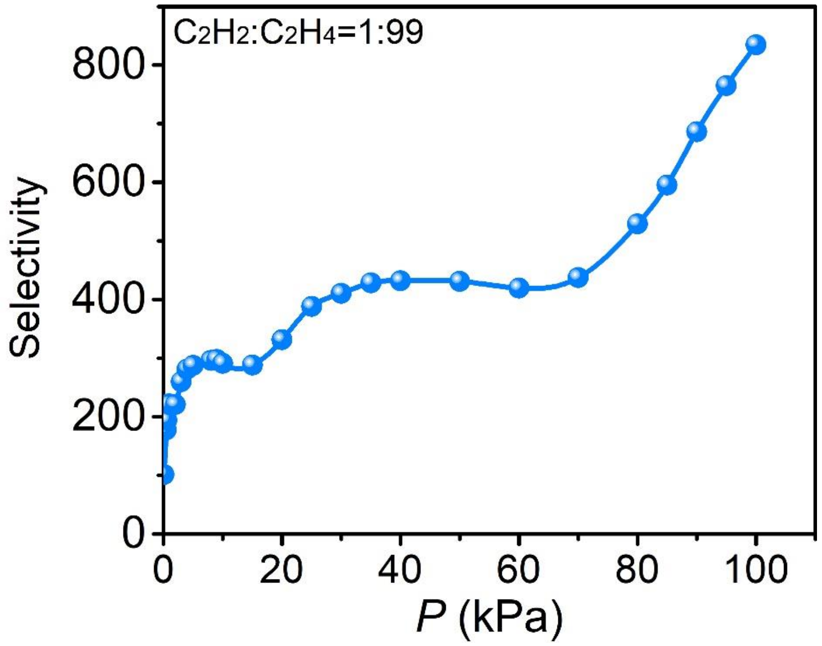

Figure S12. IAST selectivity of Co(4-DPDS)$_2$CrO$_4$ for a 1/99 C_2H_2/C_2H_4 mixture.

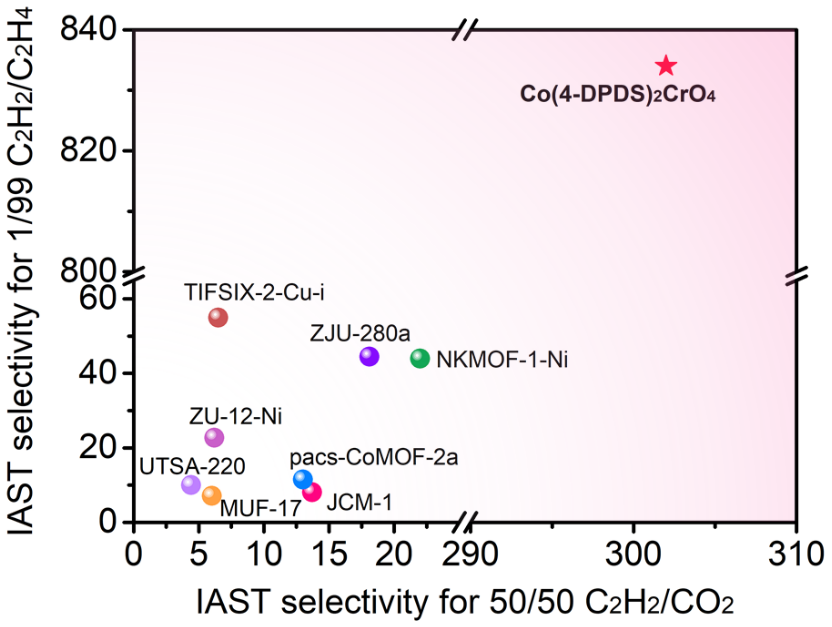

Figure S13. Comparison of 1/99 C_2H_2/C_2H_4 and 50/50 C_2H_2/CO_2 selectivity of Co(4-DPDS)$_2$CrO$_4$ with those of other materials reported under ambient conditions.

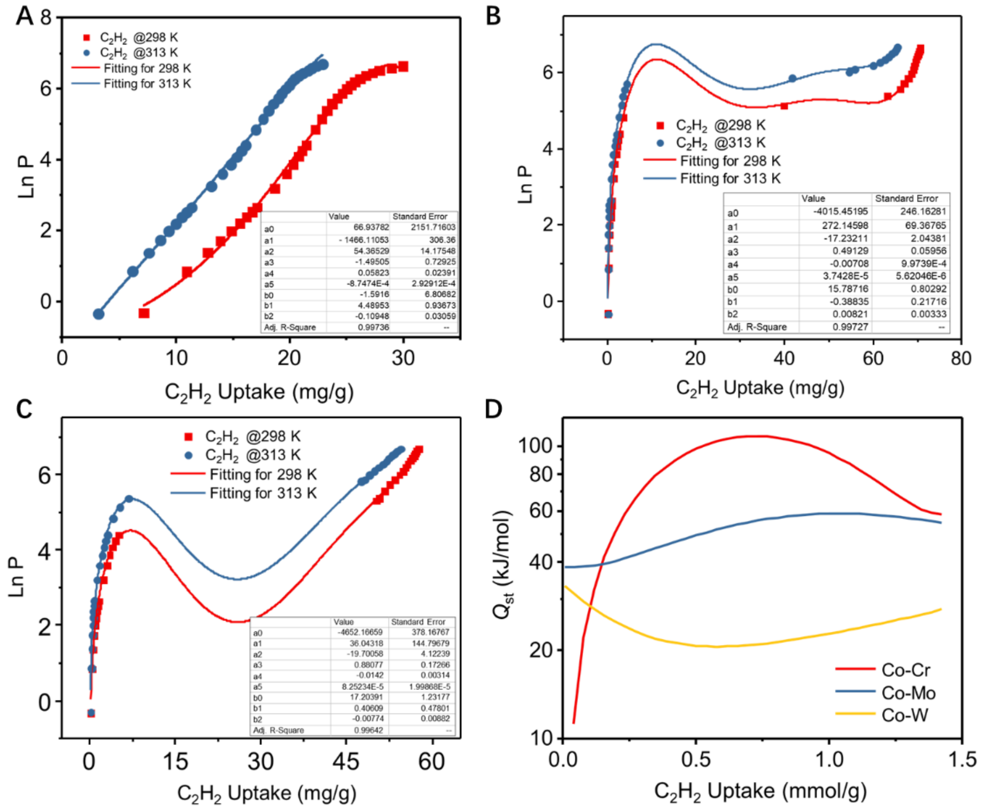

Figure S14. The Virial fittings for C_2H_2 isotherms at 298 and 313 K on (A) Co(4-DPDS)$_2$CrO$_4$, (B) Co(4-DPDS)$_2$MoO$_4$ and (C) Co(4-DPDS)$_2$WO$_4$. (D) The calculated Q_{st} curves for C_2H_2 on three materials.

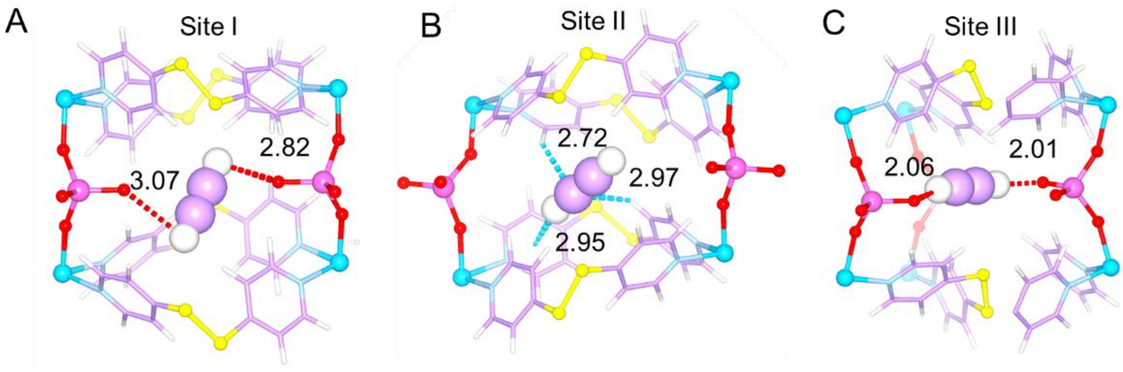

Figure S15. The calculated adsorption binding sites in Co(4-DPDS)$_2$WO$_4$ for C_2H_2. (A) site I, (B) site II and (C) site III in Co(4-DPDS)$_2$WO$_4$.

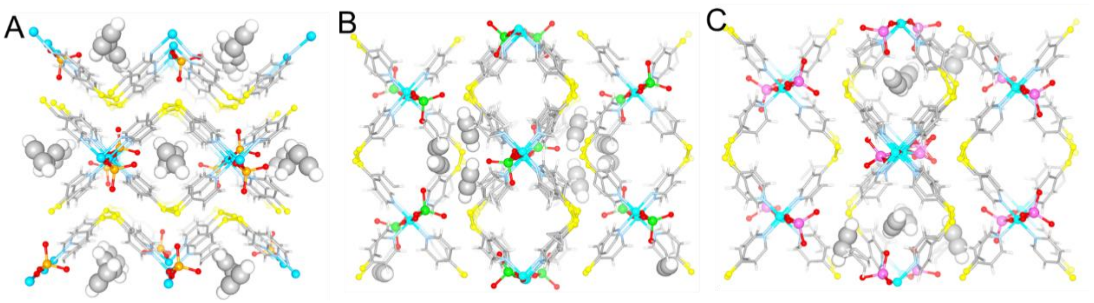

Figure S16. The crystal structure of (A) Co(4-DPDS)$_2$CrO$_4$⊃C_2H_2; (B) Co(4-DPDS)$_2$MoO$_4$⊃C_2H_2; (C) Co(4-DPDS)$_2$WO$_4$⊃C_2H_2. There is disorder in the skeleton of Co(4-DPDS)$_2$MoO$_4$ and Co(4-DPDS)$_2$WO$_4$.

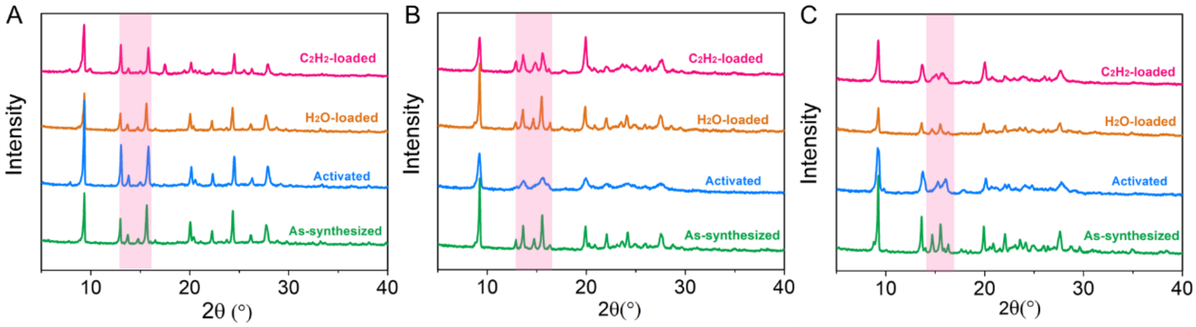

Figure S17. PXRD patterns of (A) Co(4-DPDS)$_2$CrO$_4$, (B) Co(4-DPDS)$_2$MoO$_4$, (C) Co(4-DPDS)$_2$WO$_4$.

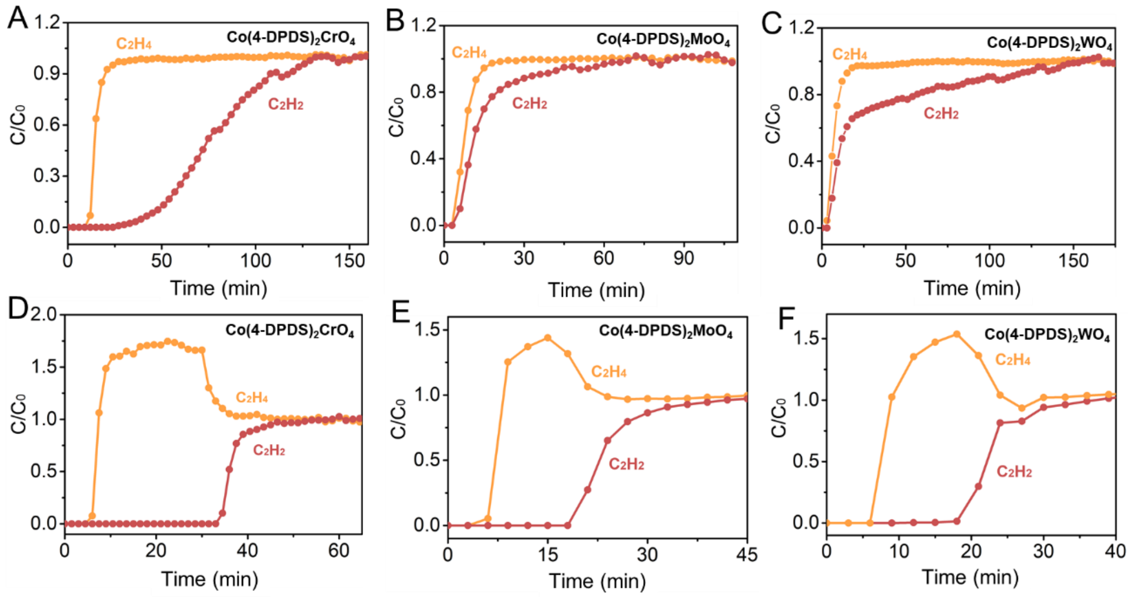

Figure S18. The 1/99 and 50/50 C_2H_2/C_2H_4 experimental breakthrough performance at 298 K. (A) (D) Co(4-DPDS)$_2$CrO$_4$; (B) (E) Co(4-DPDS)$_2$MoO$_4$; (C) (F) Co(4-DPDS)$_2$WO$_4$.

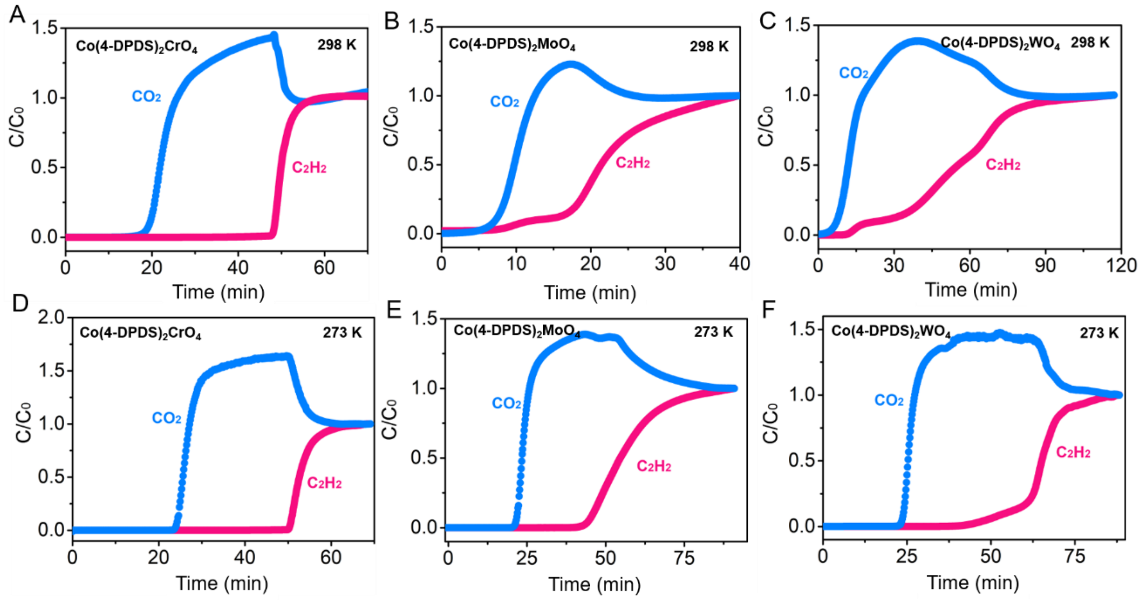

Figure S19. The 50/50 C_2H_2/CO_2 experimental breakthrough performance at 298, 273 K, repspectively. (A) (D) Co(4-DPDS)$_2$CrO$_4$; (B) (E) Co(4-DPDS)$_2$MoO$_4$; (C) (F) Co(4-DPDS)$_2$WO$_4$.

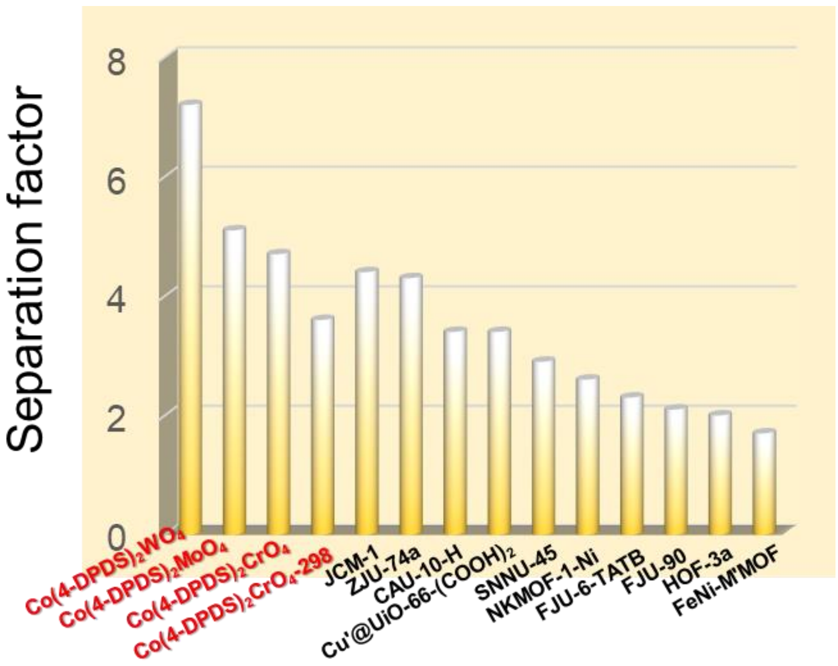

Figure S20. Comparison of C_2H_2/CO_2 (50:50, *v*:*v*) separation factor obtained by experimental breakthrough curves for Co(4-DPDS)$_2$MO$_4$ at 273 K and other top-performing materials.

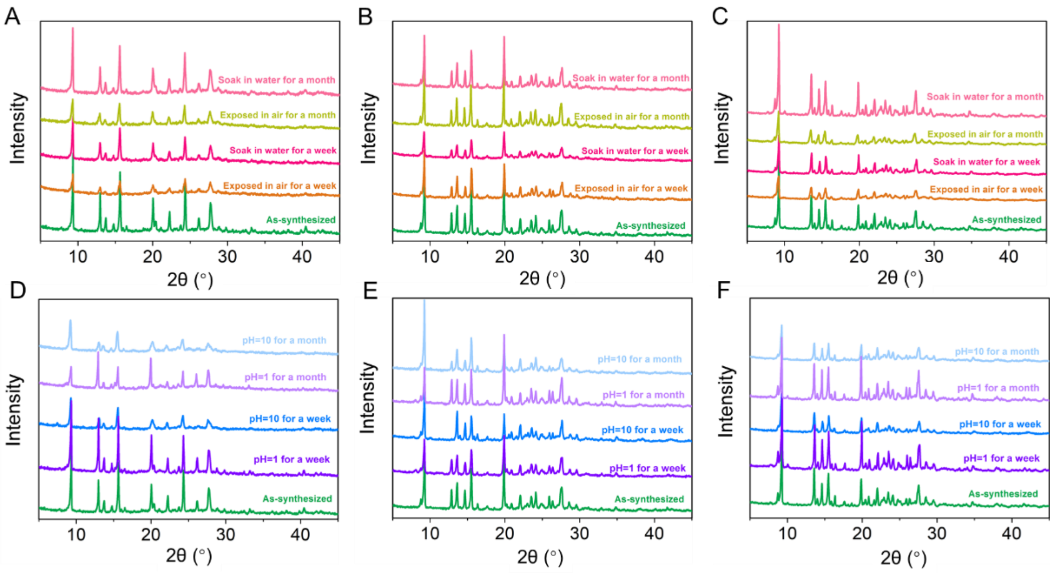

Figure S21. PXRD pattern of air and water stability of the material. (A) Co(4-DPDS)$_2$CrO$_4$, (B) Co(4-DPDS)$_2$MoO$_4$, (C) Co(4-DPDS)$_2$WO$_4$; PXRD pattern of chemical stability in acid/base solution. (D) Co(4-DPDS)$_2$CrO$_4$, (E) Co(4-DPDS)$_2$MoO$_4$, (F) Co(4-DPDS)$_2$WO$_4$.

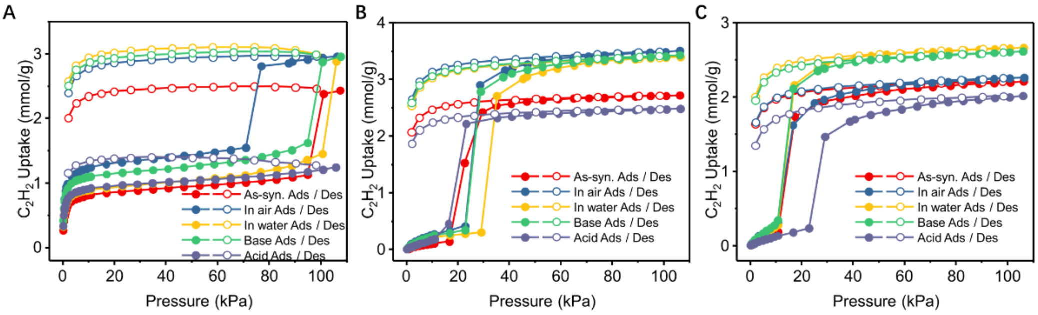

Figure S22. The C_2H_2 isotherms at 298 K on (A) Co(4-DPDS)$_2$CrO$_4$, (B) Co(4-DPDS)$_2$MoO$_4$ and (C) Co(4-DPDS)$_2$WO$_4$ before and after stability tests.

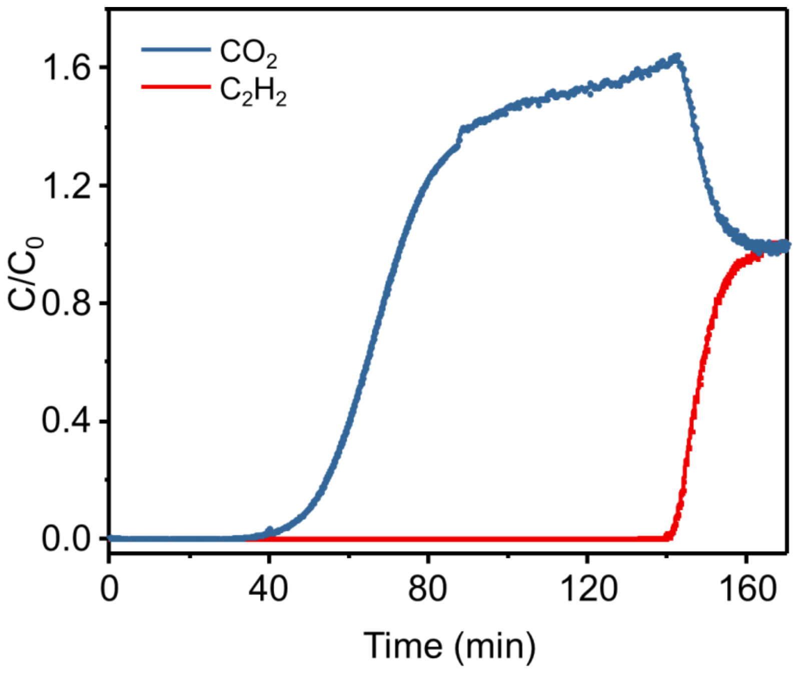

Figure S23. Experimental column breakthrough curves for a wet 50/50 C_2H_2/CO_2 mixture with a total flow of 0.6 mL·min^{-1} in an absorber bed packed with Co(4-DPDS)$_2$CrO$_4$ at 298 K and 1 bar under 100% humidity.

Table S1. Crystallographic data and structure refinement result for as-synthesized samples

Identification code	Co(4-DPDS)$_2$CrO$_4$	Co(4-DPDS)$_2$MoO$_4$	Co(4-DPDS)$_2$WO$_4$
Chemical formula	$C_{20}H_{16}CoCrN_4O_4S_4$	$C_{20}H_{16}CoMoN_4O_4S_4$	$C_{20}H_{16}CoWN_4O_4S_4$
Formula weight	615.54	659.48	747.39
Temperature	190 K	190(2) K	296(2) K
Crystal system	monoclinic	monoclinic	monoclinic
Space group	C2/m	P 21/c	P 21/c
Unit cell dimensions	a = 23.9748(11) Å<br>α = 90°	a = 21.4347(11) Å<br>α = 90°	a = 21.398(3) Å<br>α = 90°
	b = 10.6665(6) Å<br>β = 124.174(2)°	b = 13.7125(8) Å<br>β = 117.924(2)°	b = 13.7382(17) Å<br>β = 117.842(4)°
	c = 13.5647(8)<br>γ = 90°	c = 22.8757(12) Å<br>γ = 90°	c = 22.846(4) Å<br>γ = 90°
Volume	2869.9(3)	5940.9(6)	5938.6(15)
Z	4	8	8
Calc.density (g/cm^3)	1.425	1.475	1.672

Table S2. Crystallographic data and structure refinement result for activated samples

Identification code	Co(4-DPDS)$_2$CrO$_4$	Co(4-DPDS)$_2$MoO$_4$	Co(4-DPDS)$_2$WO$_4$
Chemical formula	$C_{20}H_{16}CoCrN_4O_4S_4$	$C_{40}H_{32}Co_2Mo_2N_8O_8S_8$	$C_{40}H_{32}Co_2W_2N_8O_8S_8$
Formula weight	615.54	1318.95	1494.77
Temperature	153 K	190 K	296 K
Crystal system	orthorhombic	monoclinic	monoclinic
Space group	Imma	P 21/n	P 21/n
Unit cell dimensions	a = 10.6916(16) Å<br>α = 90°	a = 21.4347(11) Å<br>α = 90°	a = 21.995(3) Å<br>α = 90°
	b = 13.576(2) Å<br>β = 90°	b = 13.7125(8) Å<br>β = 117.924(2)°	b = 13.5429(16) Å<br>β = 119.731(4)°
	c = 19.663(3) Å<br>γ = 90°	c = 22.8757(12) Å<br>γ = 90°	c = 22.021(3) Å<br>γ = 90°
Volume	2854.1(8)	5940.9(6)	5696.1(13)
Z	4	4	4
Calc.density (g/cm^3)	1.432	1.475	1.743

Table S3. Crystallographic data and structure refinement results of Co(4-DPDS)$_2$MO$_4$ after C_2H_2 adsorbates loaded

Identification code	Co(4-DPDS)$_2$CrO$_4$ ⊃C_2H_2	Co(4-DPDS)$_2$MoO$_4$⊃ C_2H_2	Co(4-DPDS)$_2$WO$_4$⊃ C_2H_2
Chemical formula	$C_{22}H_{18}CrN_4CoO_4S_4$	$C_{42}H_{34}Mo_2N_8Co_2O_8S_8$	$C_{42}H_{34}W_2N_8Co_2O_8S_8$
Formula weight	641.57	1344.99	760.18
Temperature	296 K	193 K	193(2) K
Crystal system	Orthorhombic	Orthorhombic	Orthorhombic
Space group	P b c n	C 2c 2	C 2c 2
Unit cell dimensions	a = 19.393(3) Å α= 90°	a =21.5988(19) Å α= 90°	a = 21.6877(17) Å α= 90°
	b =10.8075(17) Å β= 90°	b=39.670(4) Å β= 90°	b = 39.348(3) Å β= 90°
	c =10.8075(17) Å γ= 90°	c=13.6737(11) Å γ= 90°	c =13.661(1) Å γ= 90°
Volume	5645.7(16)	11716.0(18)	11658.0(16)
Z	8	8	8
Calc.density (g/cm^3)	1.510	1.525	1.733

Table S4. Summary of BET surface areas, adsorption uptakes at 298 K and 1 bar, and C_2H_2/CO_2 uptake ratio at 1.0/1.0 bar and 298 K for MOFs with C_2H_2/CO_2 separation performance

MOFs	$S_{BET}(m^2 \cdot g^{-1})$	C_2H_2 uptake($cm^3 \cdot g^{-1}$)	CO_2 uptake($cm^3 \cdot g^{-1}$)	Uptake ratio	S_{ads}
MUF-17[3]	247	67.4^a	56.2^a	1.2	6.0^a
JNU-1[4]	818	63.0	51.0	1.2	3.0
SNNU-45[5]	1007	134.0	97.4	1.4	4.5
ZJU-196a[6]	N.A.	83.5^b	8.5^b	9.8	N.A.
ZJU-74[7]	694	85.7^b	69.0^b	1.2	36.5^b
ZJU-280[8]	810	106.0^b	71.0^b	1.5	18.1
$Cu_2(pzdc)_2(pyz\text{-}NH_2)$[9]	103	41.2	4.6	9.0	119.0
JCM-1[10]	550	75.0	38.0	2.0	13.7
FJU-90a[11]	1572	180.0	103.0	1.8	4.3
UTSA-74a[12]	830	107.0	71.0	1.5	9.0
UTSA-300a[13]	311	68.9	3.3	21.2	743.0
UTSA-300(Cu)[14]	358	102.0	14.0	7.3	N.A.
DiCRO-4-Ni-i[15]	398	43.0	23.0	1.9	13.9
$Ni_3(COOH)_6$[16]	289	94.0	68.0	1.4	22.0
$Cu_2(ade)_2(PA)_2$[17]	401	49.0	33.7	1.5	5.7
FeNi-M'MOF[18]	382	96.0	60.0	1.6	24.0
NKMOF-1-Ni[19]	382	61.0	51.1	1.2	30.0
TCuCl[20]	167	67.2	44.8	1.5	16.9
TCuBr[20]	173	62.7	44.8	1.4	9.5
FJU-6-TATB[21]	1306	110.0^b	58.0^b	1.9	3.1^b
BSF-3-Co[22]	458	80.4	47.0	1.7	16.3
CPM-733-dps[23]	1883	234.0	124.0	1.9	N.A.
SIFSIX-Cu-TPA[24]	1330	185.9	107.5	1.7	5.3
SIFSIX-21-Ni[25]	871	90.7	29.0	3.1	7.8
ATC-Cu[26]	600	112.0	90.0	1.2	53.6
CAU-10-H[27]	183	89.8^b	60.0^b	1.5	4.0
MFM-188[28]	2568	232.0	120.0	1.9	N.A.
Cu^I@UiO-66-$(COOH)_2$ [29]	302	51.5	20.2	2.6	185.0
**$Co(4\text{-}DPDS)_2CrO_4$**	92	54.4/57.6	10.5/14.6	5.2/3.9	302.0
**$Co(4\text{-}DPDS)_2MoO_4$**	65	60.8/62.9	7.2/12.5	8.5/5.1	/
**$Co(4\text{-}DPDS)_2WO_4$**	64	49.6/56.0	9.0/11.8	5.5/4.8	/

^{a}At 293 K. ^{b}At 296 K. ^{c}At 273 K. ^{d}IAST selectivity at 0.15 bar for (1: 1, *v*/*v*) C_2H_2/CO_2.

Table S5. The fitting parameters for Dual Site Langmuir-Freundlich isotherm model of Co(4-DPDS)$_2$CrO$_4$

Adsorbates	q_A (mmol·g^{-1})	b_A (kPa^{-vA})	n_A dimensionless	q_B (mmol·g^{-1})	b_B (kPa^{-vB})	n_B dimensionless	R^2
C_2H_2 (298 K)	1.4390	0.0000	77.1491	1.2401	0.8095	0.3799	0.99
C_2H_4 (298 K)	0.1361	0.9127	1.0884	0.5367	0.0151	0.5479	0.9999
CO_2 (298 K)	0.5000	0.0007	1.1961	0.425	0.1169	0.9842	0.9999

References

[1] a) K. S. Walton, D. S. Sholl, *AIChE J.* **2015**, *61*, 2757-2762; b) A. L. M. a. J. M. PRAUSNITZ, *AIChE J.* **1995**, *11*, 121-126.

[2] a) M. D. Segall, P. J. D. Lindan, M. J. Probert, C. J. Pickard, P. J. Hasnip, S. J. Clark, M. C. Payne, *Journal of Physics Condensed Matter* **2002**, *14*, 2717-2744; b) P. J. Meza-Morales, A. Santana-Vargas, M. C. Curet-Arana, *Adsorption* **2015**, *21*, 533-546.

[3] T. Q. Omid, B. Ravichar, G. T. Shane, *Chem Mater* **2019**, *31*, 4919−4926.

[4] H. Zeng, M. Xie, Y. L. Huang, Y. Zhao, X. J. Xie, J. P. Bai, M. Y. Wan, R. Krishna, W. Lu, D. Li, *Angew. Chem. Int. Ed. Engl.* **2019**, *58*, 8515-8519.

[5] Y. P. Li, Y. Wang, Y. Y. Xue, H. P. Li, Q. G. Zhai, S. N. Li, Y. C. Jiang, M. C. Hu, X. Bu, *Angew. Chem. Int. Ed. Engl.* **2019**, *58*, 13590-13595.

[6] L. Zhang, K. Jiang, L. Li, Y. P. Xia, T. L. Hu, Y. Yang, Y. Cui, B. Li, B. Chen, G. Qian, *Chem. Commun. (Camb)* **2018**, *54*, 4846-4849.

[7] J. Pei, K. Shao, J. X. Wang, H. M. Wen, Y. Yang, Y. Cui, R. Krishna, B. Li, G. Qian, *Adv. Mater.* **2020**, *32*, e1908275.

[8] Q. Quan-Li, G. Xiao-Wen, P. Jiyan, W. Hui-Min, W. Hui, Z. Wei, L. Bin, Q. Guodong, *J. Mater. Chem. A* **2021**, *9*, 9248.

[9] L. Yang, L. Yan, Y. Wang, Z. Liu, J. He, Q. Fu, D. Liu, X. Gu, P. Dai, L. Li, X. Zhao, *Angew. Chem. Int. Ed. Engl.***2020**, *60*, 4570–4574.

[10] L. Jaechul, C. Chong Yang, K. Jaheon, K. Youngsuk, K. Nakeun, S. Younggyu, K. Kimoon, B. Tae Hyun, L. Eunsung, *Angew. Chem. Int. Ed. Engl.* **2018**, *57*, 7869 –7873.

[11] Y. Ye, Z. Ma, R. B. Lin, R. Krishna, W. Zhou, Q. Lin, Z. Zhang, S. Xiang, B. Chen, *J. Am. Chem. Soc.* **2019**, *141*, 4130-4136.

[12] F. Luo, C. Yan, L. Dang, L. Wang, M. Luo, R. Krishna, W. Zhou, H. Wu, X. Dong, Y. Han, T.-L. Hu, M. O'Keeffe, R.-B. Lin, B. Chen, *J. Am. Chem. Soc.* **2016**, *138*, 5678-5684.

[13] R. B. Lin, L. Li, H. Wu, H. Arman, B. Li, R. G. Lin, W. Zhou, B. Chen, *J. Am. Chem. Soc.* **2017**, *139*, 8022-8028.

[14] J. Wang, Y. Zhang, P. Zhang, J. Hu, R. B. Lin, Q. Deng, Z. Zeng, H. Xing, S. Deng, B. Chen, *J. Am. Chem. Soc.* **2020**, *142*, 9744-9751.

[15] H. S. Scott, M. Shivanna, A. Bajpai, D. G. Madden, K. J. Chen, T. Pham, K. A. Forrest, A. Hogan, B. Space, J. J. Perry Iv, M. J. Zaworotko, *ACS Appl. Mater. Interfaces* **2017**, *9*, 33395-33400.

[16] Z. Ling, J. Ke, Z. Jun, P. Jiyan, S. Kai, C. Yuanjing, Y. Yu, L. Bin, C. Banglin, Q. Guodong, *Acs Sustain. Chem. Eng.* **2018**, *7*, 1667−1672.

[17] H. Li, H. Bonduris, X. Zhang, Y. Ye, A. Alsalme, R.-B. Lin, Z. Zhang, S. Xiang, B. Chen, *J. Solid. State. Chem.* **2020**, *284*, 121209.

[18] J. Gao, X. Qian, R. B. Lin, R. Krishna, H. Wu, W. Zhou, B. Chen, *Angew. Chem. Int. Ed. Engl.* **2020**, *59*, 4396-4400.

[19] Y. L. Peng, T. Pham, P. Li, T. Wang, Y. Chen, K. J. Chen, K. A. Forrest, B. Space, P. Cheng, M. J. Zaworotko, Z. Zhang, *Angew. Chem. Int. Ed. Engl.* **2018**, *57*, 10971-10975.

[20] S. Mukherjee, Y. He, D. Franz, S. Q. Wang, W. R. Xian, A. A. Bezrukov, B. Space, Z. Xu, J. He, M. J. Zaworotko, *Chemistry* **2020**, *26*, 4923-4929.

[21] L. Liu, Z. Yao, Y. Ye, Y. Yang, Q. Lin, Z. Zhang, M. O'Keeffe, S. Xiang, *J. Am. Chem. Soc.* **2020**,

142, 9258-9266.

[22] Y. Zhang, J. Hu, R. Krishna, L. Wang, L. Yang, X. Cui, S. Duttwyler, H. Xing, *Angew. Chem. Int. Ed. Engl.* **2020**, *59*, 17664-17669.

[23] Y. Wang, X. Jia, H. Yang, Y. Wang, X. Chen, A. N. Hong, J. Li, X. Bu, P. Feng, *Angew. Chem. Int. Ed. Engl.* **2020**, *59*, 19027-19030.

[24] H. Li, C. Liu, C. Chen, Z. Di, D. Yuan, J. Pang, W. Wei, M. Wu, M. Hong, *Angew. Chem. Int. Ed. Engl.* **2021**, *60*, 1 – 7.

[25] N. Kumar, S. Mukherjee, N. C. Harvey-Reid, A. A. Bezrukov, K. Tan, V. Martins, M. Vandichel, T. Pham, L. M. van Wyk, K. Oyekan, A. Kumar, K. A. Forrest, K. M. Patil, L. J. Barbour, B. Space, Y. Huang, P. E. Kruger, M. J. Zaworotko, *Chem* **2021**, *7*, 1–14.

[26] Z. Niu, X. Cui, T. Pham, G. Verma, P. C. Lan, C. Shan, H. Xing, K. A. Forrest, S. Suepaul, B. Space, A. Nafady, A. M. Al-Enizi, S. Ma, *Angew. Chem. Int. Ed. Engl.* **2021**, *60*, 5283-5288.

[27] B. Chen, J. Pei, H. M. Wen, X. W. Gu, Q. L. Qian, Y. Yang, Y. Cui, B. Li, G. Qian, *Angew. Chem. Int. Ed. Engl.* **2021**, *60*, 25068–25074.

[28] M. Florian, S. Ivan da, H. A. S. Nada, L. E. Timothy, S. Mathew, G. W. G. Harry, F. P. Stewart, M. Pascal, Y. Sihai, S. Martin, *Nat.Commun.* **2017**, *8*, 14085.

[29] L. Zhang, K. Jiang, L. Yang, L. Li, E. Hu, L. Yang, K. Shao, H. Xing, Y. Cui, Y. Yang, B. Li, B. Chen, G. Qian, *Angew. Chem. Int. Ed. Engl.* **2021**, *60*, 15995-16002.
